# Structural and evolutionary dissection of NADPH‐binding motifs in NADPH‐preferring ene‐reductases

**DOI:** 10.1002/pro.70521

**Published:** 2026-03-18

**Authors:** Bianca Kerschbaumer, Eva M. Frießer, Silvia Wallner, Gustav Oberdorfer, Michael Friess, Rolf Breinbauer, Peter Macheroux, Aleksandar Bijelic

**Affiliations:** ^1^ Institute of Biochemistry Graz University of Technology Graz Austria; ^2^ BioTechMed‐Graz Graz Austria; ^3^ Institute of Organic Chemistry Graz University of Technology Graz Austria

**Keywords:** ancestral sequence reconstruction, ene‐reductase, NADPH binding, old yellow enzyme, protein structure–function

## Abstract

Ene‐reductases (ERs) catalyze nicotinamide‐dependent, stereoselective reductions of activated C=C bonds. While their catalytic chemistry and applications are well‐explored, cosubstrate (NAD(P)H) binding remains poorly understood. Most ERs strongly prefer NADPH despite lacking canonical dinucleotide‐binding folds and instead employ flexible loop motifs. We recently elucidated the NADPH‐binding mode of the NADPH‐preferring ER *Solanum lycopersicum* OPR3 (*Sl*OPR3), identifying four key residues (R283/R343/Y364/R366) that form two motifs: a 2′‐phosphate (2′‐P)‐binding site (R343/Y364/R366) and a loop 6 (L6)‐mediated adenine clamp (R283/R343). Guided by this model, we analyzed the conservation of these motifs across 51 NADPH‐preferring ERs from different Old Yellow Enzyme (OYE) classes by multi‐sequence alignment and homology modeling. Analyses revealed a class‐dependent distribution: class‐II ERs predominantly conserve the OPR3‐like motifs, whereas other classes employ alternative mechanisms, including dimerization‐induced modes. Functional dissection of *Sl*OPR3 through mutagenesis, kinetics, and crystallography established a functional hierarchy of the motif elements, indicating that R343 and R366 are indispensable for NADPH binding in OPR3‐like ERs, while the adenine clamp acts as a conformation‐sensitive affinity tuner. Ancestral sequence reconstruction revealed the stepwise and convergent assembly of motif elements, culminating in the complete motif set in plant, fungal, and cyanobacterial lineages. Our findings delineate (i) a strict functional hierarchy of NADPH‐binding residues in OPR3‐like ERs, (ii) alternative binding solutions in other OYE classes, and (iii) a convergent evolutionary trajectory, advancing the fundamental understanding of NADPH binding in NADPH‐preferring ERs and offering a modular framework to predict NADPH preference in ERs.

## 1 INTRODUCTION

Ene‐reductases (ERs), members of the flavin mononucleotide (FMN)‐dependent Old Yellow Enzyme family (OYE, EC 1.6.99.1), catalyze the stereoselective reduction of activated C=C bonds at the expense of nicotinamide cofactors (NADH or NADPH). This asymmetric reduction is among the most widely applied biocatalytic transformations, establishing ERs as the state‐of‐the‐art for biocatalytic alkene reduction (Stuermer et al., 2007). While the catalytic mechanism and potential biocatalytic applications of ERs are well documented in the literature, little is known about their cosubstrate‐binding mechanisms.

Unlike most nucleotide‐dependent oxidoreductases, ERs lack canonical cosubstrate‐binding folds, such as the Rossmann fold (Cahn et al., 2017). Instead, they employ mobile and poorly conserved active‐site loops to anchor their cosubstrates. Despite this lack, ERs typically display a marked preference or even dependence on one cosubstrate—most often NADPH (Brigé et al., 2006; Chaparro‐Riggers et al., 2007; Elegheert et al., 2017; Fitzpatrick et al., 2003; Knaus et al., 2016; Kubata et al., 2002; Sellés Vidal et al., 2018).

Sequence similarity across ERs is low (<15%) despite the enzymes sharing the typical (βα)_8_‐barretopology (Fox & Karplus, 1994; Iorgu et al., 2019). Due to such pronounced sequence divergence, particularly within solvent‐exposed loops that shape the FMN‐ and nicotinamide‐binding regions, the OYE family has been divided into six classes (I–VI) (Damada and Fraaije, 2024; Robescu et al., 2020, 2022). Classes I and II encompass classical OYEs, such as many NADPH‐dependent ERs, which are primarily found in yeasts and bacteria, whereas Classes III and IV feature more divergent sequences, often with distinct active site loops and oligomeric states. Classes V and VI are more recently characterized and contain further structural variations compared to the other classes. This classification reflects the evolutionary adaptability of ERs and highlights how cosubstrate recognition may vary significantly among closely related homologs.

Because ERs lack dedicated nucleotide‐binding folds, elucidating the molecular basis of their cosubstrate specificity is crucial—not only to understand how enzymes evolve cosubstrate preference beyond canonical binding motifs, but also for biocatalysis, where NADH offers superior stability and regeneration systems and significantly lower cost than NADPH. However, the low sequence similarity and high plasticity of the active‐site architecture complicate the identification of common structural determinants governing cosubstrate binding in ERs.

**FIGURE 1:**
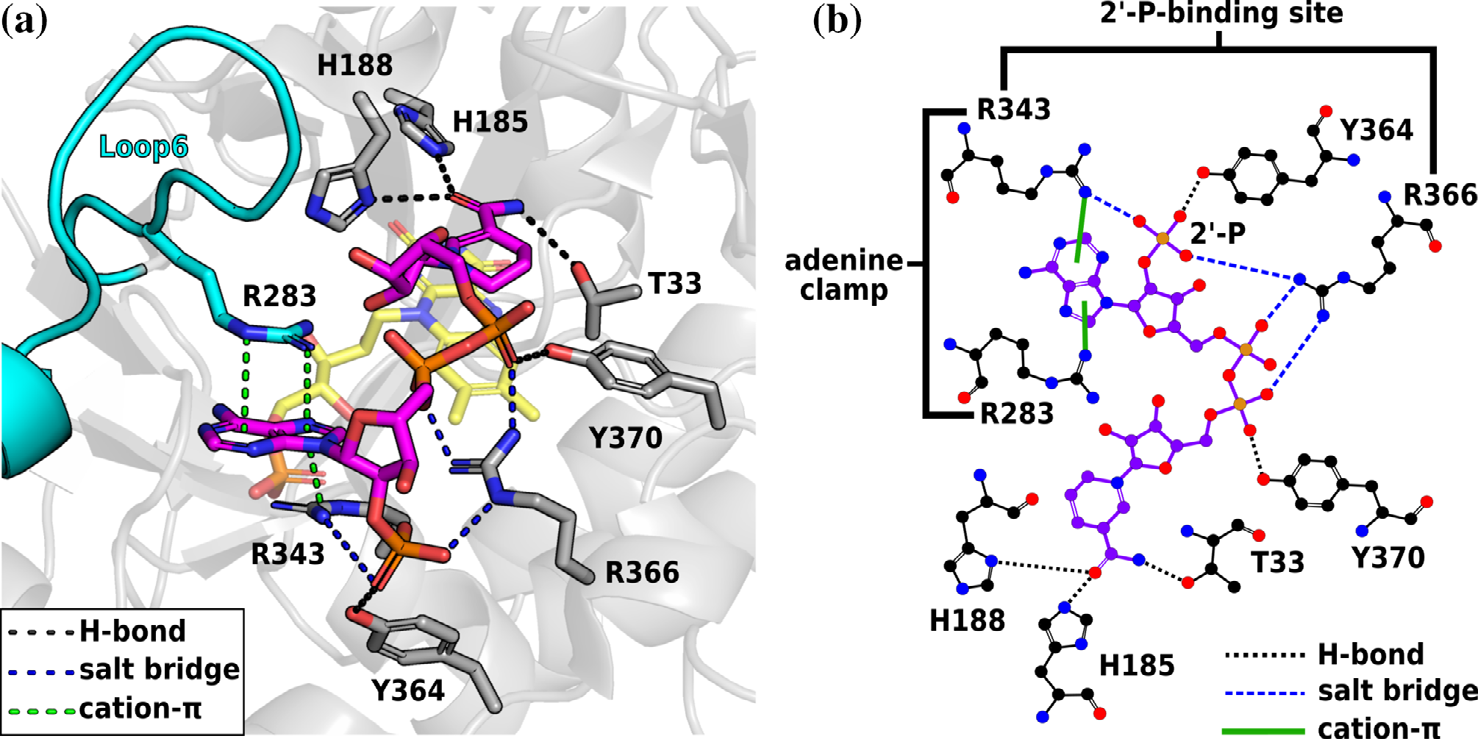
NADPH‐binding mode in *Sl*OPR3. (a) Crystal structure of the *Sl*OPR3–NADPH_4_ complex (PDB: 8QMX). NADPH_4_ is depicted as purple sticks, while the FMN cofactor is represented by yellow transparent sticks. (b) Ligplot showing the *Sl*OPR3–NADPH_4_ interactions schematically. The two composite binding motifs, 2′‐P‐binding site (R343/Y364/R366) and the adenine clamp (R343/R283), are highlighted.

In this regard, we previously identified such determinants in the NADPH‐preferring ER 12‐oxophytodienoic acid reductase 3 from *Solanum lycopersicum* (*Sl*OPR3) (Kerschbaumer, Totaro, et al., 2024). A large conformational change in loop 6 (L6) creates a transient NADPH‐binding site in *Sl*OPR3 (Figure 1). At this site, two composite motifs act in concert: a 2′‐phosphate binding site (2′‐P site) formed by R343/Y364/R366 to anchor the 2′‐P group and an L6‐mediated adenine clamp (R283/R343) to lock the adenine group within a cation–π–cation sandwich.

In contrast, NADH‐preferring morphinone reductase stabilizes the adenosine tail against a β‐hairpin (β‐HP) flap opposite L6, involving mainly less specific hydrophobic contacts (Pudney et al., 2007). However, recognition and binding of the nicotinamide moiety are conserved among ERs.

Despite these insights from single systems, it remains unclear how conserved such binding motifs are across the OYE family. As prior work has focused mainly on individual enzymes, the modular principles and class‐dependency underlying NADPH selectivity are insufficiently defined.

Here, we address this gap by (i) surveying NADPH‐preferring ERs across OYE classes for conservation of OPR3‐like residues (R283/R343/Y364/R366) and composite motifs (2′‐P site/adenine clamp), (ii) determining the relative functional contribution of each element and of L6 conformation via mutagenesis, kinetics, and X‐ray crystallography in *Sl*OPR3, and (iii) employing ancestral sequence reconstruction to place these features in an evolutionary context and to assess how widely an OPR3‐like mechanism applies.

## 2 RESULTS AND DISCUSSION

### 2.1 Class‐dependent conservation of OPR3‐like NADPH‐binding motifs

To assess the conservation of the NADPH‐binding residues (R283/R343/Y364/R366) and the associated composite motifs (2′‐P site/adenine clamp) identified in *Sl*OPR3, we curated a list of NADPH‐preferring ERs. For this, we searched the literature for ERs that were experimentally shown to prefer NADPH at least twice over NADH (Table S1). After assigning each ER to an OYE class, we included only ERs from the major OYE classes (I–III) in our analysis (*N* = 43), as the remaining classes were underrepresented. Across the 43 ERs (*N*_classI_ = 17, *N*_classII_ = 16, and *N*_classIII_ = 10), 21 ERs carry the complete OPR3‐like signature. Residue numbering follows the *Sl*OPR3 sequence nomenclature throughout.

**FIGURE 2:**
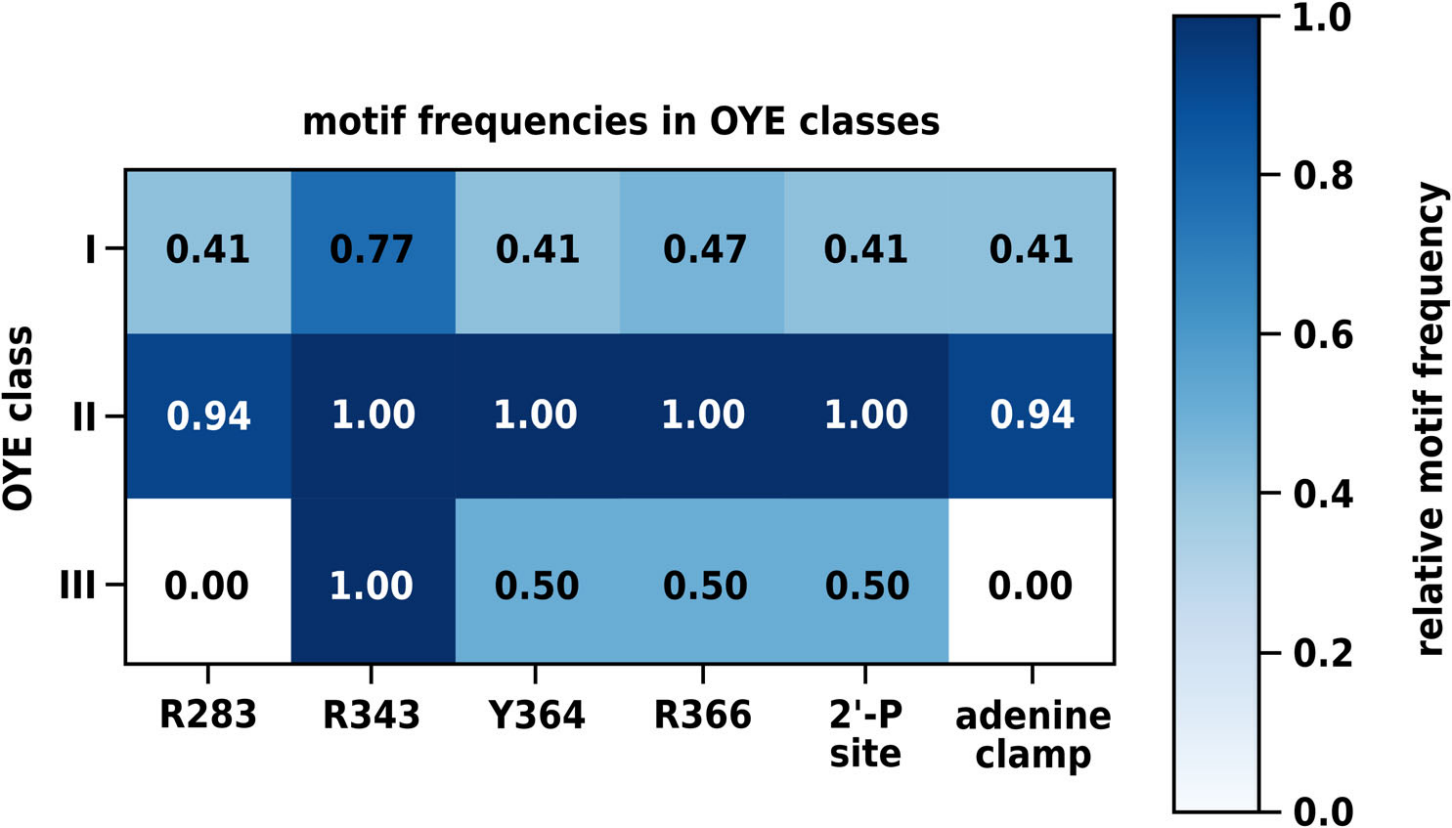
Heatmap showing relative frequencies of the OPR3‐like NADPH‐binding motifs across NADPH‐preferring ERs. Number of ERs found for each class: *N*_classI_ = 17, *N*_classII_ = 16, and *N*_classIII_ = 10.

Analysis of the sequences showed that motif usage is class‐dependent (Figure 2). Class II (fungal OYEs) nearly universally retains the full OPR3 motif set, with the 2′‐P site conserved in all (*p* < 0.001) and the adenine clamp in 94% (*p* < 0.001) of cases. Class III (thermophilic‐like OYEs) lacks R283 entirely and thus cannot form the adenine clamp (*p* < 0.001), but retains the 2′‐P site in ~50% of cases (*p* = 0.28). Class I (classical OYEs from plants and bacteria) exhibits an intermediate pattern, with both the 2′‐P site and adenine clamp present in 41% of members (*p* < 0.05 and *p* = 0.23). Notably, the 50% frequency of the 2′‐P site in class III and the 41% frequency of the adenine clamp in class I were statistically not significant (due to small sample size).

We next examined whether NADPH preference, expressed as log_10_ (NADPH/NADH) activity ratio (see Methods section), correlates with NADPH signature completeness. Presence of the 2′‐P site correlates only weakly with higher NADPH preference (Δ ≈ 0.25, *p* = 0.38), whereas the adenine clamp shows essentially no effect (Δ ≈ −0.08; *p* = 0.93). Importantly, reported NADPH preferences have been derived from different experimental approaches (see Table S1), limiting direct comparability.

Overall, the results underscore that motif conservation segregates by OYE class but fails to correlate with NADPH preference. The high frequency of R343 throughout classes is due to its involvement in FMN binding, as most ERs have a positively charged residue at this position to stabilize the flavin's ribityl phosphate group. Class III employs adenine clamp‐independent binding modes. In members retaining the 2′‐P site, the motif is formed by dimerization, as the residues corresponding to Y364 and R366 are contributed by the C‐terminal domain of an adjacent protomer, defining a C‐terminal YxR‐motif. The involved arginine (arginine finger) was first described in YqjM and plays a key role in substrate binding (Kitzing et al., 2005). In contrast, most class III members lacking the 2′‐P site—as exemplified by XenA—feature a tryptophan (tryptophan finger) at the equivalent position, which, together with other aromatic residues, forms a tunnel that π‐stacks and stabilizes the cosubstrate's adenine ring (Spiegelhauer et al., 2010). This highlights at least two mechanistic solutions for NADPH binding within class III, with the arginine‐finger‐based mechanism being similar to the OPR3‐like one. Notably, all plant and nearly all fungal ERs in our dataset retain the complete OPR3 motif, suggesting evolutionary conservation of the *Sl*OPR3‐type binding mode across plant and fungal lineages.

### 2.2 Functional hierarchy of motif elements in *Sl*OPR3

**FIGURE 3:**
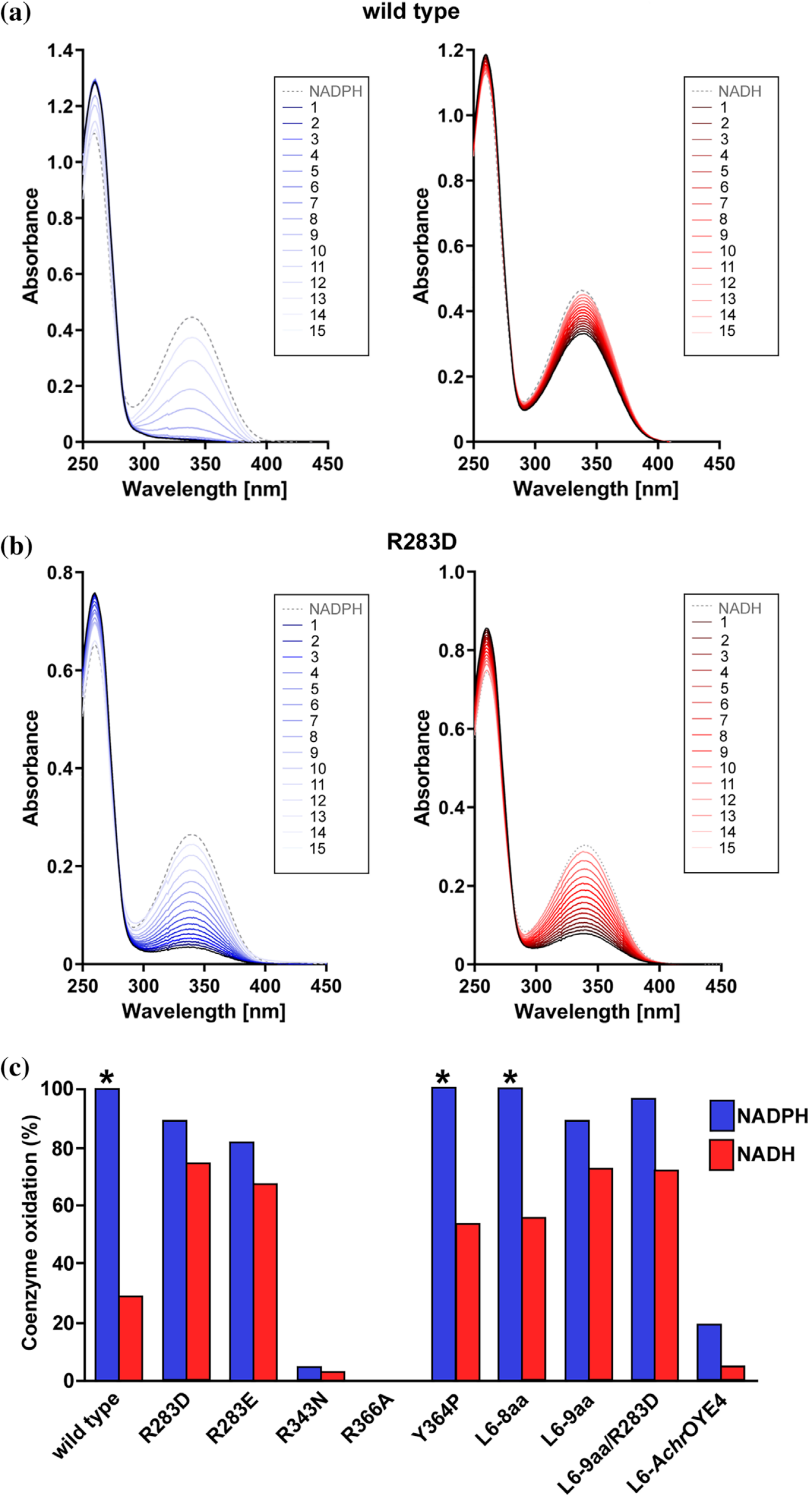
Cosubstrate acceptance assay. (a) Acceptance assay of the wild type, monitoring the decrease of the characteristic 340‐nm band of NAD(P)H. (b) Acceptance assay of variant R283D. Assay results for the remaining variants are presented in Figure S1. (c) Graphic summary of the acceptance assays of all constructs. In (a) and (b), the spectrum of each of the 15 cycles is shown (see legend). The asterisks (*) in (c) indicate complete NAD(P)H oxidation. Among fully oxidizing constructs, the reaction rates were as follows: Y364P > wild type > > 9aa.

Because several highly NADPH‐preferring ERs lack a complete OPR3‐like signature, we asked whether all residues of the motif contribute equally to NADPH binding. To this end, *Sl*OPR3 variants targeting R283, R343, Y364, R366, and L6 were generated and analyzed for NADPH acceptance (Figure 3 and Figure S1). Substituting R343 or R366 nearly abolished activity with both cosubstrates, with R366 exerting the most severe effect. By contrast, substitution of R283 only moderately reduced NADPH activity but increased NADH activity substantially. Targeting L6 produced more nuanced outcomes: swapping L6 with that from the NADH‐preferring ER from *Achromobacter* sp. JA81 (L6‐*Achr*OYE4) caused a greater loss of NADPH activity than loop truncation (variants L6‐9aa and ‐8aa). Interestingly, only the L6 truncation variants caused a substantial increase in NADH activity while the activity of the swap variant was comparable to that of the wild type. Among the truncation variants, L6‐8aa, in which L6 was truncated from 18 to 8 residues, was less active with NADPH than variant 9aa, in which L6 was truncated to 9 residues, retaining residue R283. Additional substitution of R283 in variant 9aa (L6‐9aa/R283D) further compromised NADPH activity while slightly improving NADH activity. In contrast, substituting Y364 had almost no effect on NADPH consumption, highlighting its minor role in NADPH binding. Notably, most variants—except R343N, R366A, and L6‐AchrOYE4—exhibited significantly greater NADH acceptance than the wild type, offering promising starting points for cosubstrate‐switching campaigns in *Sl*OPR3.

To obtain a more detailed view, we determined steady‐state kinetic parameters using NADPH as cosubstrate and 2‐methylmaleimide as substrate (Table S2). While the wild type displayed a high turnover (*k*_cat_ = 17.2 s^−1^), the variants were strongly impaired, with the loop variants showing a ~8–12‐fold reduction in turnover (*k*_cat_ = 1.5–2.1 s^−1^) and the R283 substitutions a ~ 34–39‐fold reduction (*k*_cat_ = 0.44–0.50 s^−1^).

To disentangle cosubstrate binding (*K*_d_) from hydride‐transfer activity (*k*_red_), we also measured stopped‐flow kinetics of the reductive half‐reaction (FMN reduction by NAD(P)H) (Table 1 and Figure S2). Due to negligible activity, no kinetic parameters could be determined for R343N and R366A. In general, the kinetic results corroborated those from the acceptance assay; however, the variant‐induced impairments were significantly more pronounced in the kinetic data. For instance, substituting R283 in L6‐9aa left *k*_red_ essentially unchanged (2.59 vs. 2.54 s^−1^) but weakened NADPH binding ~9‐fold (*K*_d_ = 76 vs. 671 μM), supporting a primary role of R283 in productive NADPH binding. Interestingly, the loop swap induced a second kinetic phase, with both phases characterized by very weak NADPH binding (*K*_d_ in mM range). Consistent with the acceptance assay, most variants (except L6‐*Achr*OYE4) showed increased NADH reactivity relative to wild type.

**TABLE 1:** Pre‐steady state kinetics of *Sl*OPR3 wild type and variants.

Enzyme	NADPH		NADH	
	*k*_red_ (s^−1^)	*K*_d_ (μM)	*k*_red_ (s^−1^)	*K*_d_ (μM)
Wild type^a^	17.0 ± 0.39	28 ± 4.0	0.99 ± 0.01	1397 ± 60
R283D	2.25 ± 0.02	603 ± 20	2.38 ± 0.02	1157 ± 47
R283E	2.12 ± 0.02	746 ± 26	1.77 ± 0.02	1216 ± 60
L6‐8aa	1.38 ± 0.03	106 ± 11	1.34 ± 0.05	532 ± 60
L6‐9aa	2.59 ± 0.08	76 ± 11	0.87 ± 0.01	531 ± 24
L6‐9aa/R283D	2.54 ± 0.03	671 ± 23	2.82 ± 0.03	766 ± 26
L6‐*Achr*OYE4^b^	2.55 ± 0.03 (1)	3678 ± 128	3.17 ± 0.10 (1)	7874 ± 820
	0.78 ± 0.02 (2)	1452 ± 104	0.41 ± 0.02 (2)	869 ± 105

^a^ Kinetic data for *Sl*OPR3 wild type were taken from reference (Kerschbaumer, Totaro, et al., 2024).

^b^ In this variant, flavin reduction kinetics were biphasic; the parameters for the faster and slower phases are indicated by (1) and (2), respectively. Values are provided as mean ± SD.

Together, our data establish a functional hierarchy of motif elements in NADPH binding: R366 ≈ R343 > L6 (swap) > R283 > L6 (truncation) > Y364. Thus, R343 and R366 are indispensable for NADPH binding in OPR3‐like ERs, while L6/R283 constitute a tunable adenine‐capture module.

### 2.3 Structural effects of motif manipulations on NADPH binding

To rationalize our kinetic data, we solved the crystal structures of the *Sl*OPR3 variants R283D, R283E, L6‐9aa, L6‐8aa, and L6‐*Achr*OYE4 in complex with the unreactive NADPH analog NADPH_4_. Unfortunately, we failed to obtain crystals for the variants R343N and L6‐9aa/R283D. Variant R366A was crystallized without a cosubstrate in the active site (but with a peripherally bound cosubstrate), corroborating its abolished cosubstrate activity (Figure S3). Data and refinement statistics for all crystal structures, including the PDB entry IDs, are summarized in Tables S3–S8.

#### 2.3.1 Structural effects of the R283 substitutions

The crystal structure of R283D–NADPH_4_ forms an intriguing crystallographic tetramer including a “semi‐self‐inhibitory” dimer (Kerschbaumer, Macheroux, & Bijelic, 2024) and a dimer with an NADPH_4_‐bound protomer (Figure S4). For more information on the overall structure, please see the Supporting Information.

**FIGURE 4:**
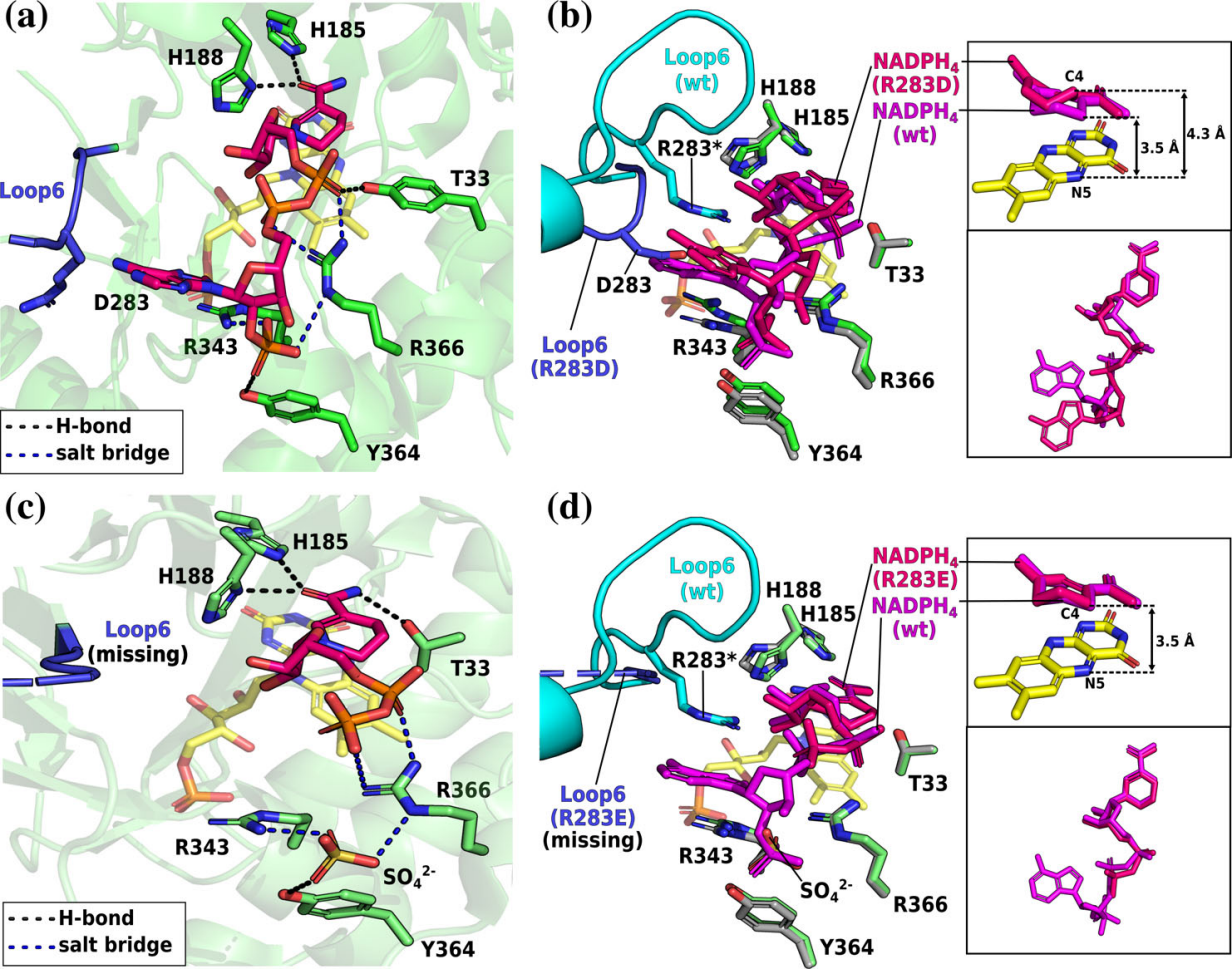
Crystal structures of the *Sl*OPR3 R283D– and R283E–NADPH_4_ complexes. (a) NADPH_4_ binding in R283D. (b) Superimposition of the NADPH_4_‐bound R283D (green) and wild‐type (gray) structures. The insets show the superimposition of the cosubstrates. (c) NADPH_4_ binding in R283E. Note that L6 is completely missing in this structure due to disorder. (d) Superimposition of the NADPH_4_‐bound R283E (green) and wild‐type (gray) structures. The insets show the superimposition of the cosubstrates. The variants are displayed as green cartoons and sticks, with their L6 highlighted in blue; the wild type is shown as a gray cartoon and sticks, with its L6 highlighted in cyan. The cosubstrates of the variants and the wild type are shown as dark pink and purple sticks, respectively. FMN is depicted as yellow sticks.

Both R283D and R283E prevented L6 from completing the NADPH‐binding site (Figure 4a,c). In R283D, the introduced aspartate forms a bidentate salt bridge with R343, displacing L6 into the adenine‐binding site of the wild type, thereby spatially narrowing the active‐site entrance (Figure 4b). This disturbance forces the adenine ring into the bulk solvent, destabilizing it. The binding mode of the nicotinamide moiety is conserved among ERs, placing the cosubstrate above the FMN's isoalloxazine ring (C4_cosubstrate_–N5_FMN_ distance <4.0 Å). However, in R283D, the nicotinamide undergoes an out‐of‐plane bending, placing the cosubstrate's C4 atom slightly away from the flavin cofactor (C4_cosubstrate_–N5_FMN_ distance = 4.3 Å; 3.5 Å in wild type) and thereby impairing the hydride transfer (Fraaije & Mattevi, 2000).

In R283E, no strong salt bridge was formed between E283 and R343, but L6 (residues E283–A302 are missing) and the cosubstrate's adenine tail are highly disordered, with a sulfate ion occupying the 2′‐P site (Figure 4c). Despite this, the resolved portion of NADPH_4_ aligned closely with that in the wild type, forming a catalytically competent complex and explaining the less severe kinetic impairment compared to R283D (Figure 4d). In contrast to R283D, R283E seems to affect NADPH binding by inducing a higher degree of disorder in L6. However, we cannot exclude competition between the cosubstrate's 2′‐P‐group and sulfate, which was used as a crystallization additive.

The structures highlight the dual role of R283 in maintaining an NADPH‐competent L6 conformation and participating in the adenine clamp.

#### 2.3.2 Structural effects of truncating L6

Crystal structures of the truncation variants (including the cosubstrate‐free enzymes) showed that L6 truncation did not affect the cosubstrates' overall fold but still displayed disordered L6 regions.

**FIGURE 5:**
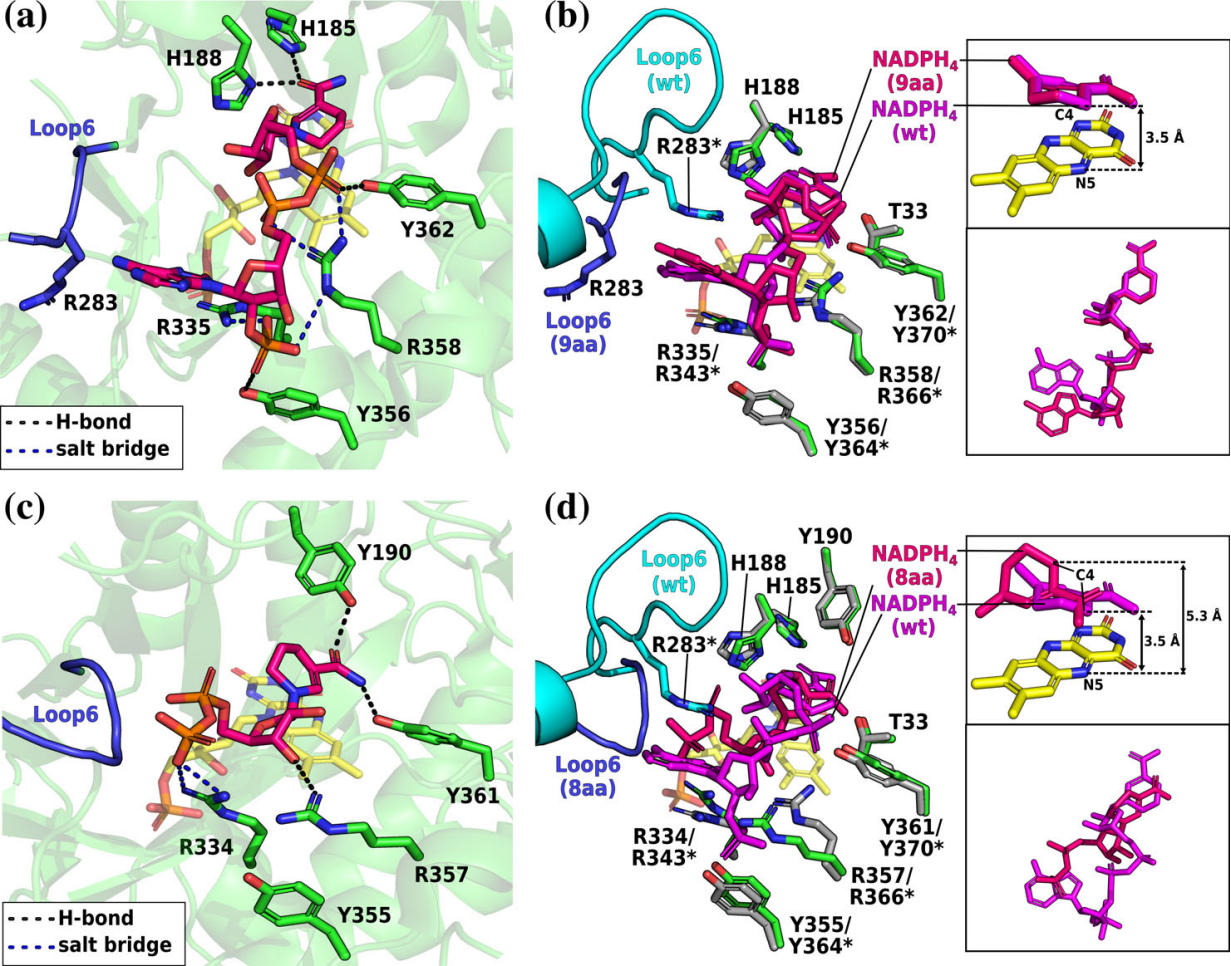
Crystal structures of the *Sl*OPR3 L6‐9aa– and L6‐8aa–NADPH_4_ complexes. (a) NADPH_4_ binding in L6‐9aa. (b) Superimposition of the NADPH_4_‐bound L6‐9aa (green) and wild‐type (gray) structures. The insets show the superimposition of the cosubstrates. (c) NADPH_4_ binding in L6‐8aa. (d) Superimposition of the NADPH_4_‐bound L6‐8aa (green) and wild‐type (gray) structures. The insets show the superimposition of the cosubstrates. The coloring scheme is the same as in Figure 4. Asterisks (*) indicate the corresponding sequence numbering of the wild type.

In the L6‐9aa–NADPH_4_ structure, the cosubstrate binds in a configuration resembling that in the wild‐type structure, with proper 2′‐P anchoring but failure to form the adenine clamp, as R283 points away from the active site (Figure 5a). This R283 conformation leaves the cosubstrate's adenine ring solvent‐exposed and thus unstabilized. The structure suggests that the truncated L6 is too short and constrained to form the adenine clamp. Nevertheless, the nicotinamide moiety binds in a catalytically competent configuration, explaining the highest NADPH activity of L6‐9aa among the variants (Figure 5b). As shown by our kinetic data, additional R283 substitution further destabilizes the complex, mirroring the above‐described effect of the R283D substitution and the critical tuning role of R283.

In contrast, L6‐8aa binds NADPH_4_ in a distorted geometry, with the nicotinamide group significantly shifted (C4_cosubstrate_–N5_FMN_ distance = 5.3 Å) and the cosubstrate tail highly disordered, resulting in a catalytically unproductive binding (Figure 5c,d). The cosubstrate's backbone is located very close to L6, preventing the 2′‐P group from interacting with the 2′‐P site (Figure 5d). The crystal structure corroborates the kinetic defect of L6‐8aa via formation of an unproductive complex (lowest k_red_ value).

Overall, both truncations prevent L6 from forming the native NADPH‐binding motif. 9aa still binds the cosubstrate's 2′‐P group and appears to provide sufficient space for (less stable) adenine binding through R283‐mediated opening of L6. In contrast, due to the lack of R283, 8aa binds the cosubstrate in a catalytically unproductive manner.

#### 2.3.3 Structural effects of the loop swap

**FIGURE 6:**
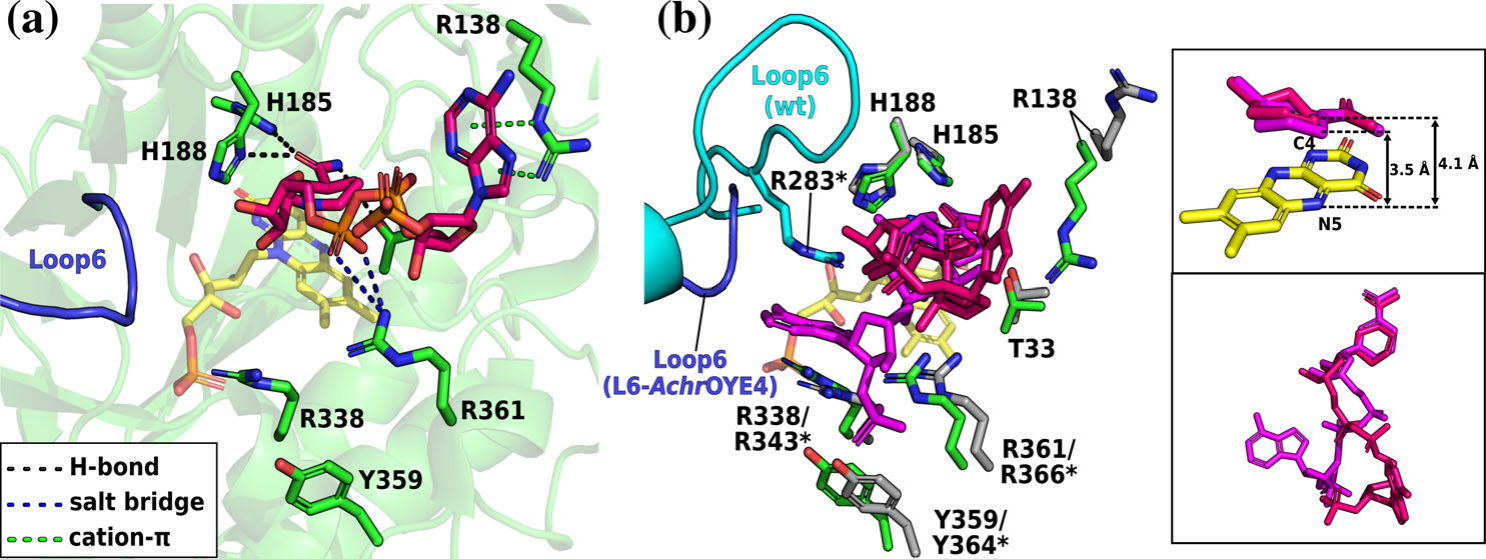
Crystal structures of the L6‐*Achr*OYE4–NADPH_4_ complex. (a) NADPH_4_ binding in L6‐*Achr*OYE4. (b) Superimposition of the NADPH_4_‐bound L6‐*Achr*OYE4 (green) and wild‐type (gray) structures. The insets show the superimposition of the cosubstrates. The coloring scheme is the same as in Figure 4. Asterisks (*) indicate the corresponding sequence numbering of the wild type.

In L6‐*Achr*OYE4, the chimeric loop is highly disordered (5 of 12 residues could be modeled) but does not affect the enzyme's overall structure, except for some partial unfolding of the α‐helix following L6. The crystal structure contains two chains; one with a completely modeled cosubstrate (despite diffuse electron density) and one with a highly disordered 2′‐P‐adenosine tail. In the first chain, the loop swap induced a striking relocation of the NADPH_4_ adenine tail (Figure 6a). Instead of engaging the L6 region, the cosubstrate's adenosine moiety binds to the β‐HP site—located opposite L6—and is stabilized by R138, which undergoes a marked conformational change toward the cosubstrate compared with its position in the wild type (Figure 6b). However, the 2′‐P group is highly solvent‐exposed and unstabilized. The chimeric loop composition, enriched in acidic and nonpolar residues (AEADWDDAPDMP vs. wild type: QPRYVAYGQTEAGRLGS), appears to repel the negatively charged 2′‐P group, diverting NADPH_4_ away from the L6 region. In the second chain, the 2′‐P‐adenosine tail is completely disordered because R138 does not engage the cosubstrate.

Collectively, our crystal structures show that the NADPH‐binding motif in *Sl*OPR3 (wild type) forms an electrostatically (arginine residues) and geometrically (closed L6 conformation) tailored microenvironment for a negatively charged recognition motif (2′‐P). R366 emerges as the dominant determinant, as it not only interacts with 2′‐P but also with the phosphate backbone, thereby positioning the cosubstrate and enabling adenine capture. Thus, the presence of this residue can partially rescue NADPH activity in other variants. In contrast, L6 and R283 modulate access to the site and adjust adenine stabilization, while Y364 is dispensable.

### 2.4 Structural effects of motif manipulations on NADH binding

We also determined the crystal structures of the above variants in complex with NADH_4_ to elucidate structural features underlying their improved NADH binding compared to the wild type. Data and refinement statistics for all crystal structures, including the PDB entry IDs, are summarized in Tables S3–S8.

#### 2.4.1 Structural effects of the R283 substitutions

**FIGURE 7:**
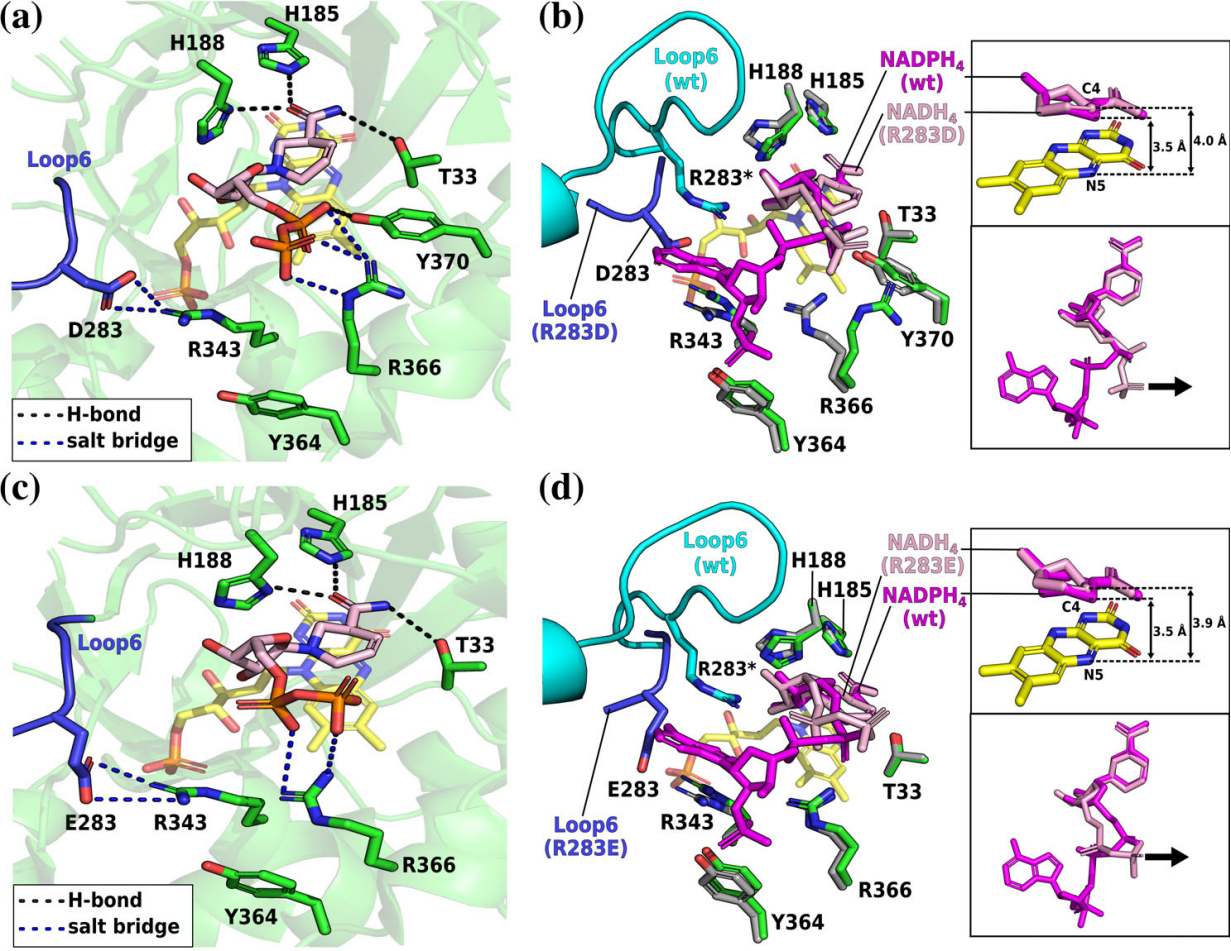
Crystal structures of the *Sl*OPR3 R283D– and R283E–NADH_4_ complexes. (a) NADH4 binding in R283D. (b) Superimposition of the NADH_4_‐bound R283D (green) and NADPH_4_‐bound wild‐type (gray) structures. The insets show the superimposition of the cosubstrates. (c) NADH_4_ binding in R283E. (d) Superimposition of the NADPH_4_‐bound R283E (green) and NADPH_4_‐bound wild‐type (gray) structures. The insets show the superimposition of the cosubstrates. The coloring scheme is the same as in Figure 4. The arrows in the inset indicate that the tails of NADH_4_ are oriented toward the β‐HP flap.

In the R283D–NADH_4_ structure, L6 and the cosubstrate's adenosine tail are highly disordered. R283 forms a monodentate salt bridge with R343 as in the R283D–NADPH_4_ structure (Figure 7a). The nicotinamide moiety adopts the wild‐type binding mode but shows an out‐of‐plane shift (C4_cosubstrate_–N5_FMN_ distance = 4.1 Å), precluding an ideal charge‐transfer complex (Figure 7b). The pyrophosphate backbone interacts with R366 (ion pair) and Y370 (H‐bond). Notably, R366 adopts a distinct rotamer pointing toward the β‐HP region, reorienting the pyrophosphate accordingly.

In the R283E–NADH_4_ complex, L6 and the cosubstrate's adenosine tail are also highly disordered (Figure 7c). The nicotinamide of NADH_4_ binds as observed in the R283D–NADH_4_ structure (C4_cosubstrate_–N5_FMN_ distance = 3.9 Å). Again, the pyrophosphate interacts electrostatically with R366 and orients toward the β‐HP flap (Figure 7d). In contrast to D283, E283 forms only a weak salt bridge with R343, as judged by its poor electron density.

Both variants fail to accommodate NADH_4_ within the L6 region; instead, the cosubstrate's tail shifts toward the β‐HP flap, where it also cannot be properly accommodated. The improved NADH kinetics of the variants could be explained by the fact that, in the wild type (PDB: 8QN3), the pyrophosphate of NADH_4_ interacts with R283, locking the cosubstrate in an unproductive orientation, whereas in the variants, the absence of this interaction allows greater flexibility for the cosubstrate to adopt catalytically productive poses.

#### 2.4.2 Structural effects of truncating L6

**FIGURE 8:**
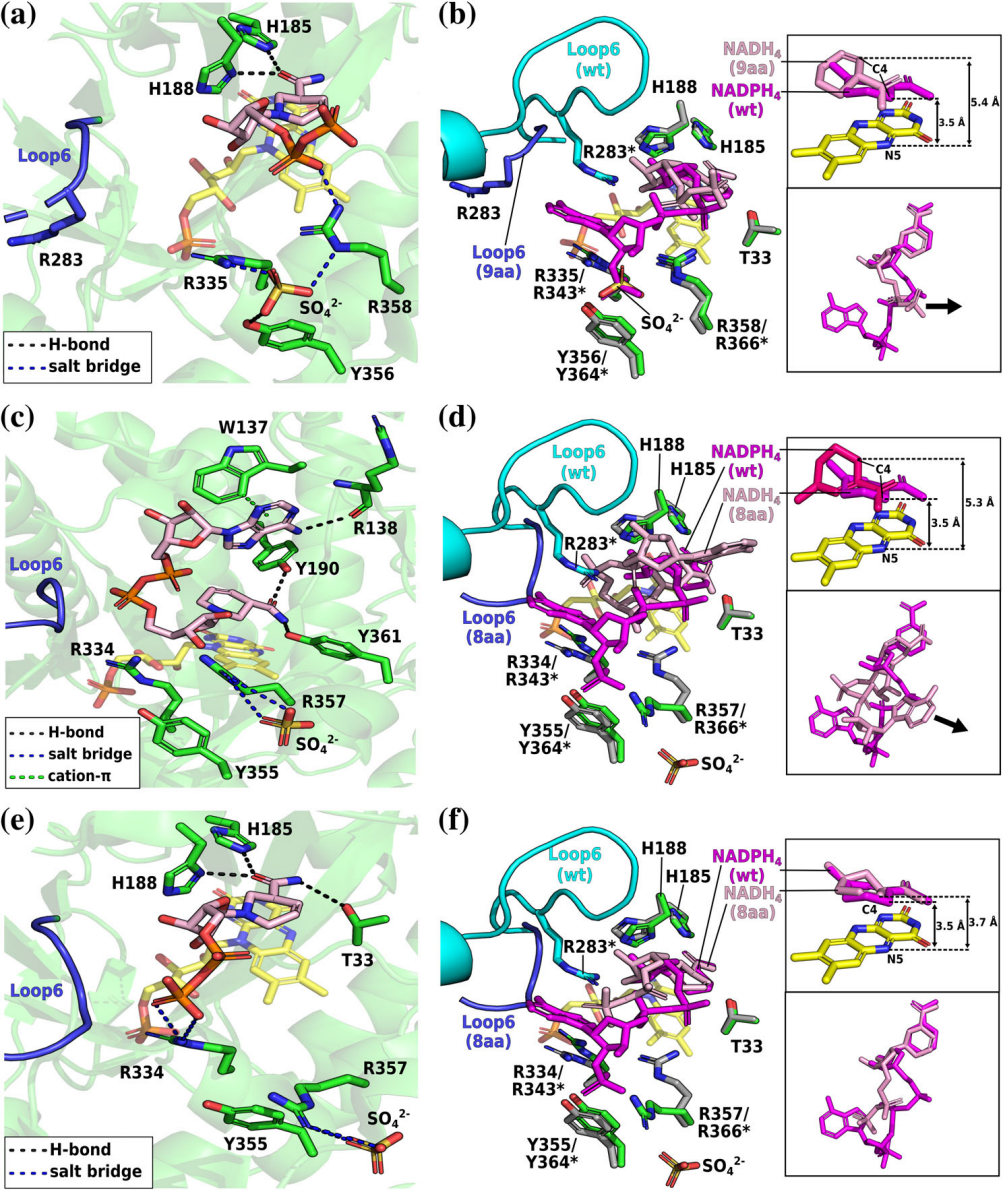
Crystal structures of the *Sl*OPR3 L6‐9aa– and L6‐8aa–NADH_4_ complexes. (a) NADH_4_ binding in L6‐9aa. (b) Superimposition of the NADH_4_‐bound L6‐9aa (green) and NADPH_4_‐bound wild‐type (gray) structures. The insets show the superimposition of the cosubstrates. (c) NADH_4_ binding to L6‐8aa in conformation 1. (d) Superimposition of the structure shown in (c) (green) and the NADPH_4_‐bound wild‐type (gray) structure. (e) NADH_4_ binding to L6‐8aa in conformation 2. (f) Superimposition of the structure shown in (e) (green) and the NADPH_4_‐bound wild‐type (gray) structure. The insets show the superimposition of the cosubstrates. The coloring scheme is the same as in Figure 4. The arrows in the inset indicate that the tails of NADH_4_ are oriented toward the β‐HP flap. Asterisks (*) indicate the corresponding sequence numbering of the wild type.

In the NADH_4_‐bound L6‐9aa structure, L6 and the cosubstrate's adenosine tail are highly disordered (Figure 8a). The nicotinamide moiety binds in the conserved mode, with its C4 atom positioned 4.0 Å above the flavin's N5 atom, which is at the threshold for catalytically competent complexes (Figure 8b). Unlike in the NADPH_4_‐bound structures, the pyrophosphate group orients toward the β‐HP region. The NADH_4_ tail engages only in ion‐pair interactions with R359 (R366 in wild type). As observed in the L6‐9aa–NADPH_4_ structure, R283 points away from the active site and does not contribute to cosubstrate binding, contrasting the situation in the wild type (Figure 8b).

In the L6‐8aa–NADH_4_ structure, L6 and the cosubstrate's adenosine tail are again highly disordered (Figure 8c). Unlike in other structures, the nicotinamide moiety showed diffuse electron density from multiple conformations, indicating nonspecific NADH_4_ binding in L6‐8aa. Eventually, we modeled the two major NADH_4_ conformations (conformation 1 and 2; Figure 8c,e). In conformation 1, the NADH_4_ molecule adopts a folded conformation in which its pyrophosphate backbone is located next to L6, while its adenine ring is positioned next to the β‐HP (Figure 8c,d). This contrasts with the extended conformation observed in all other NADH_4_‐bound structures. The position of the nicotinamide‐riboside moiety of NADH_4_ resembles that of NADPH_4_ in the L6‐8aa–NADPH_4_ structure, precluding hydride transfer (C4_cosubstrate_–N5_FMN_ distance = 5.3 Å, Figure 8d). The adenine ring H‐bonds the backbone amide of R138 and π‐stacks with W137. This folded pose is likely enabled by truncation‐related steric relief. In conformation 2, the nicotinamide adopts the conserved, catalytically competent pose (C4_cosubstrate_–N5_FMN_ distance = 3.7 Å; Figure 8e,f). The cosubstrate's pyrophosphate backbone adopts an extended conformation, interacting electrostatically with R334 (R343 in wild type). Unlike in our other NADH_4_‐bound structures, the β‐phosphate of NADH_4_ in L6‐8aa is located close to the L6 region, overlapping with the adenine‐binding site of the wild type (Figure 8f).

In both conformations, the NADH_4_ tail localizes near L6 rather than the β‐HP region, as observed elsewhere. Thus, L6 truncation and R283 absence reshape the active‐site entrance, profoundly affecting NADH accommodation in *Sl*OPR3.

#### 2.4.3 Structural effects of the loop swap

**FIGURE 9:**
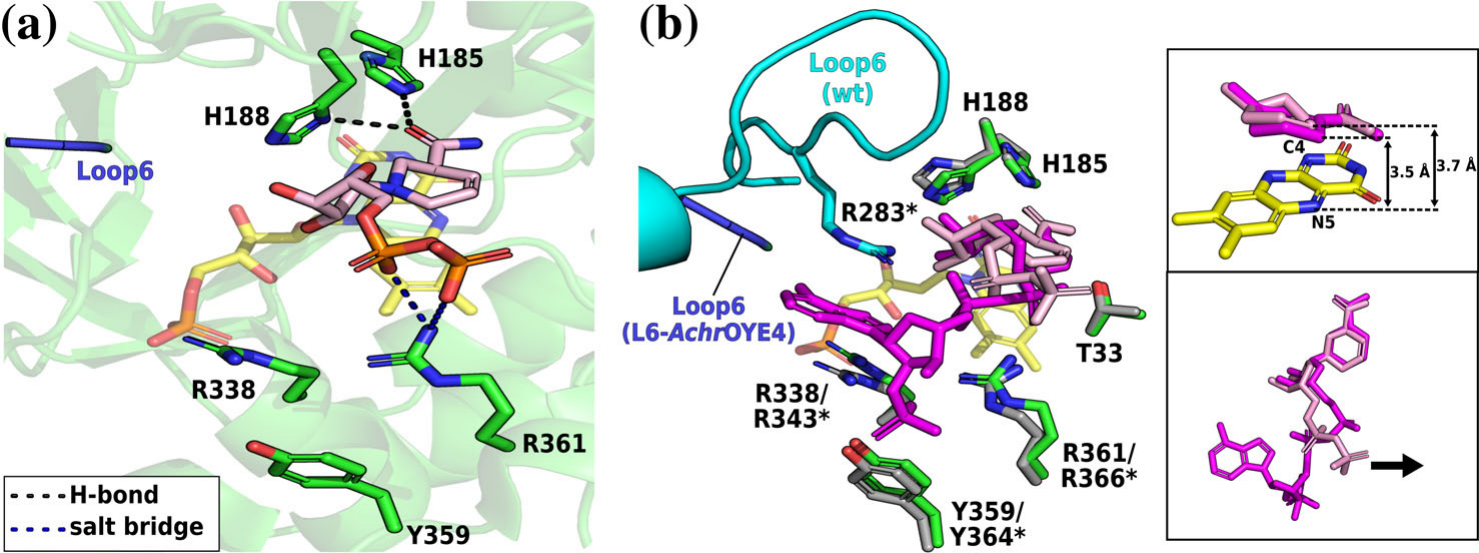
Crystal structures of the L6‐*Achr*OYE4–NADH_4_ complex. (a) NADH_4_ binding in L6‐*Achr*OYE4. (b) Superimposition of the NADH_4_‐bound L6‐*Achr*OYE4 (green) and NADPH_4_‐bound wild‐type (gray) structures. The insets show the superimposition of the cosubstrates. The coloring scheme is the same as in Figure 4. The arrows in the inset indicate that the tails of NADH_4_ are oriented toward the β‐HP flap. Asterisks (*) indicate the corresponding sequence numbering of the wild type.

Like in all other NADH_4_‐bound structures, L6 and the cosubstrate's adenosine tail are highly disordered in the L6‐*Achr*OYE4‐NADH_4_ complex (Figure 9a). The nicotinamide moiety of NADH_4_ adopts the conserved binding mode (C4_cosubstrate_–N5_FMN_ distance = 4.0 Å, Figure 9b). The cosubstrate's tail is only stabilized by ion‐ion interactions between its pyrophosphate group and R361 (R366 in wild type), leaving the remainder of the tail unstabilized. The cosubstrate's pyrophosphate orients toward the β‐hairpin region. Similar to the NADPH_4_‐bound structure, the chimeric loop likely repels the NADH tail toward the β‐hairpin.

Collectively, our NADH‐bound crystal structures reveal that—unlike NADPH—the NADH adenosine tail avoids the L6 region in all variants (except for L6‐8aa) and instead binds nonspecifically near the β‐hairpin region. Thus, the observed substantial decrease in NADPH affinity and modest increase in NADH affinity primarily reflect discrimination against NADPH rather than selection for NADH.

### 2.5 Evolutionary logic of NADPH recognition across OYE classes

To trace the evolutionary emergence and distribution of the OPR3‐like NADPH‐binding motif, we performed ancestral sequence reconstruction on 98 OYE sequences (Figure S5), focusing on the four residues (R283/R343/Y364/R366) that constitute the 2′‐P site and adenine clamp. We observed a stepwise assembly along the OPR‐like lineage: the first well‐supported node carrying the complete set is N161 (all *P* ≥ 0.98), and a convergent complete gain occurs in a cyanobacterial class‐I lineage (N143, all *P* ≥ 0.98). Immediately upstream, N160 already supports R343 and Y364 (*P* = 1.00/0.93), while R366 is ambiguous (*P* = 0.53) and R283 absent (*P* = 0.21), consistent with an anchor‐first, clamp‐later progression. The most recent common ancestor of class‐III (N102) supports the 2′‐P anchor (*P* = 1.00, 0.92, and 0.89 for R343, Y364, and R366, respectively) but lacks the adenine clamp (*P* = 0.00 for R283), matching clamp‐free recognition in extant class‐III OYEs. A more detailed analysis of the ancestral sequence reconstruction results is provided in Figure S5 and Table S9.

## 3 CONCLUSIONS

We dissected how NADPH is recognized in NADPH‐preferring ERs, highlighting both conserved and alternative solutions within the OYE family. In class‐II enzymes and plant subclades, NADPH binding resolves into two modular principles: (i) 2′‐P anchoring by an arginine‐rich microenvironment (R343/R366; Y364 auxiliary) and (ii) a dynamic, L6‐mediated adenine clamp (R283/R343). Functional analyses revealed a hierarchy of importance, with R366 and R343 being indispensable, L6 and R283 serving as conformation‐sensitive affinity modulators, and Y364 being largely dispensable. The amino acid substitutions, truncation, and loop swaps abolished the enzyme's preference for NADPH over NADH, indicating that targeting these residues and regions can substantially modulate the cosubstrate preference of OYEs.

Other OYE classes employ alternative strategies, including dimerization‐derived arginine‐finger motifs or tryptophan‐based π‐stacking, underscoring the mechanistic diversity of cosubstrate recognition. However, some of these alternative strategies seem to be similar to the OPR3‐like mode (cf. OPR3‐like motif vs. arginine‐finger motif). Ancestral reconstruction supports stepwise assembly of the signature (R343 early, R283 late) with convergent completion in plant, fungal, and cyanobacterial lineages.

Collectively, our findings delineate (i) a strict functional hierarchy of NADPH‐binding residues in OPR3‐like ERs, with R366 and R343 being indispensable for NADPH binding, (ii) mechanistically distinct NADPH‐binding modes across OYE classes, and (iii) a convergent evolution of the OPR3‐like binding motifs in plants, fungi, and cyanobacteria. This framework enables the prediction of NADPH preference based on sequence.

## 4 MATERIALS AND METHODS

### 4.1 Materials

Chemicals were purchased from Carl Roth (Karlsruhe, Germany) or Merck (Darmstadt, Germany) unless otherwise stated.

### 4.2 Literature mining for NADPH‐preferring ERs

ERs with experimentally determined NAD(P)H specificity were retrieved from Scopus and Google Scholar, using the Boolean strings (“ene‐reductase” AND “NADPH” AND “NADH”) and (“OYE” AND “NADPH” AND “NADH”). We considered only studies reporting quantitative activities with both cosubstrates. Enzymes were classified as NADPH‐ or NADH‐preferring if activity with the preferred cosubstrate was at least twofold higher than that of the alternative cosubstrate. In total, 64 ERs were compiled (51 NADPH‐ and 13 NADH‐preferring).

### 4.3 Motif–class analysis

For motif analysis, only OYE classes I–III were included because of the low sample size in the remaining classes. To evaluate whether the previously identified NADPH‐binding motifs are present in the curated ERs, we generated *Sl*OPR3‐templated SWISS models (Waterhouse et al., 2018) for each of them and superimposed them onto the crystal structure of the *Sl*OPR3–NADPH_4_ complex (PDB: 8QMX). We preferred SWISS over AlphaFold models because the strong template constraint of the former enforces a common structural frame and stable residue mapping.

Motif (R283/R343/Y364/R366/2′‐P site/adenine clamp) frequencies were compared across classes by chi‐square tests (α = 0.05). We report two‐sided *p*‐values; *χ* ^2^ statistics and effect sizes (Cramér's V) were examined to verify adequacy and are consistent with the reported *p*‐values. Analyses were performed in Python using pandas (version 1.5.3) and numpy (version 1.24) for data handling and SciPy (version 1.14.1) for statistics.

### 4.4 Design of the L6 truncation variants

The wild‐type structure of *Sl*OPR3 (PDB entry: 2HSA) was prepared for modeling in Rosetta (Leman et al., 2020) by removing all waters and relaxing the deposited coordinates using Rosetta FastRelax (Tyka et al., 2011). The resulting lowest energy structure from 100 relax runs was used as input for Rosetta Remodel (Huang et al., 2011) to rebuild L6 of *Sl*OPR3. Several blueprint files corresponding to different loop lengths were generated, and five design trajectories were run on each. The designed sequence of the lowest energy structure for each corresponding loop length was then used as input to ESM‐fold (Lin et al., 2023). The ESM‐predicted structures exhibiting the lowest all‐atom root‐mean‐square deviation (RMSD) to the redesigned structures were taken as the final models.

### 4.5 Heterologous gene expression, enzyme production, and purification

Starting from the pET21d(+)‐6xhis‐*Slopr*3 construct (Kerschbaumer, Totaro, et al., 2024), point mutations were introduced into the *Slopr3* gene using either the Q5® Site‐directed mutagenesis kit (New England Biolabs) according to the manufacturer's instructions with primer pairs generated using NEBaseChanger® or standard site‐directed mutagenesis PCR with manually designed primer pairs. Primer pairs are listed in Table S10.

L6 truncation and swap variants were generated using the standard Gibson assembly procedure (Anon, 2014). Standard PCR amplification of the pET21d(+)‐6xhis‐*Slopr3* construct was conducted using primer pairs that contained the altered, truncated, or swapped loop region(s) as overhangs (Table S10).

All constructs were transformed into chemically competent *Escherichia coli* TOP10 cells (Thermo Fisher Scientific) and verified by sequencing (LGC Genomics). All *Sl*OPR3 variants were recombinantly produced in *E. coli* BL21‐CodonPlus (DE3)‐RIL cells and purified as previously described (Kerschbaumer, Totaro, et al., 2024). For crystallization, enzymes were further purified by size‐exclusion chromatography using a Superdex 200 Increase 10/300 GL column (Cytiva) equilibrated with storage buffer.

### 4.6 Cosubstrate acceptance assay

NAD(P)H consumption was monitored by absorbance changes at 260 and 340 nm, using 1.5 μM enzyme and 50 μM cosubstrate. Spectral changes were monitored on a Specord 200 spectrophotometer (Analytik Jena) for 15 cycles á 15 s at 25°C. For analysis, the time lag between enzyme addition and the start of the measurement was considered. Spectra of 50 μM NAD(P)H (without enzyme) were recorded as reference.

### 4.7 Steady‐state kinetics

Steady‐state kinetic measurements were performed spectrophotometrically by monitoring NADPH oxidation at 340 nm using different 2‐methylmaleimide concentrations. The experiment was performed at 25°C in 50 mM Tris–HCl (pH 7.5) containing 100 μM NADPH and 250 nM *Sl*OPR3 wild‐type or variant enzyme.

Stock solutions of 2‐methylmaleimide were prepared in the above buffer, and final substrate concentrations ranged from 25 to 5000 μM.

Since cofactor reduction is the rate‐limiting step for *Sl*OPR3, *K*_M_ and *v*_max_ values for flavin reduction by NADPH were determined by fitting initial rates to the Michaelis–Menten curve in GraphPad Prism 5. All experiments were performed in triplicate.

### 4.8 Pre‐steady‐state kinetics with stopped‐flow

The pre‐steady‐state kinetic parameters of the reductive half‐reaction were determined using a stopped‐flow device (SF‐61DX2, TgK Scientific) under anoxic conditions (O_2_ = 7–9 ppm) at 25°C, as previously described (Kerschbaumer, Totaro, et al., 2024). All measurements were performed in triplicate.

### 4.9 Preparation and purification of NADPH_4_ for crystallization

The unreactive NADPH analogs 1,4,5,6‐tetrahydro‐NADPH (NADPH_4_) were synthesized, as previously described (Kerschbaumer, Totaro, et al., 2024).

### 4.10 Protein crystallization

Protein crystals of *Sl*OPR3 variants with and without NAD(P)H_4_ were grown by the vapor‐diffusion method in the hanging drop setup at 293 K. Detailed crystallization conditions for all variants are summarized in Table S11. Crystals grew within 1–3 days. To improve crystal quality, we subjected some crystals to several rounds of micro‐ and streak‐seeding using the Seed Bead Kit and Seeding Tool from Hampton Research, respectively, according to the manufacturer's instructions. Complexes with NAD(P)H_4_ were obtained by crystal soaking into reservoir solutions containing NAD(P)H_4_ for 1–60 min. To achieve the highest NAD(P)H_4_ concentration possible, we added NAD(P)H_4_ directly as powder to the soaking solutions. All crystals were cryo‐protected in reservoir solution supplemented with 50 μM NAD(P)H_4_ and 20% MPD and then vitrified in liquid nitrogen before data collection.

### 4.11 Data collection, structure solution, and refinement

X‐ray diffraction data were collected either at DESY (Hamburg, Germany) on beamline P11 or at ESRF (Grenoble, France) on beamline ID30A‐1 or ID23‐1 at 100 K. Data sets were processed and scaled using the XDS package (Kabsch, 2010). Data collection information is summarized in Tables S3–S8. The final resolution cutoff for each dataset was determined by paired refinement (Malý et al., 2020).

All structures were solved by molecular replacement using PHASER (McCoy et al., 2007) from the PHENIX suite (Adams et al., 2010). PDB entry 8QMX (chain B) was used as search model. Model building and refinement were performed with Coot (Emsley et al., 2010) and phenix.refine (Afonine et al., 2012), respectively. The presence of the cosubstrates was confirmed by polder map analysis (Liebschner et al., 2017). The final models were validated using MolProbity (Williams et al., 2018). Refinement statistics are provided in Tables S1–S6.

### 4.12 Ancestral sequence reconstruction

A non‐redundant set of 98 ER sequences was screened for obvious artifacts (truncations/frame shifts) and filtered to 20–90% pairwise identity to avoid near‐duplicates. Ancestral sequence reconstruction was performed with the FireProt‐ASR pipeline (Khan et al., 2021). FireProt performed the multiple sequence alignment internally, using *Sl*OPR3 as the query sequence. Maximum‐likelihood phylogenies were inferred under an LG + F + G model (γ‐distributed rate heterogeneity) with 1000 bootstrap replicates and a gap correction of 0.5; the tree was midpoint‐rooted. For each internal node, we recorded posterior probabilities (P). A motif residue was called present at a posterior probability ≥0.7. Robustness was confirmed across alternative thresholds and rootings. To validate our ASR class assignment, we generated an EFI‐EST (Oberg et al., 2023; Zallot et al., 2019) sequence similarity network (alignment score: 75) and clustered with MCL (inflation: 2.5), confirming overall agreement with our classification.

## AUTHOR CONTRIBUTIONS

**Bianca Kerschbaumer:** Conceptualization; investigation; writing – review and editing; methodology; validation; visualization. **Eva M. Frießer:** Investigation; validation; writing – review and editing. **Silvia Wallner:** Investigation; validation; visualization; writing – review and editing; writing – original draft. **Gustav Oberdorfer:** Investigation; formal analysis; writing – review and editing. **Michael Friess:** Investigation; writing – review and editing. **Rolf Breinbauer:** Investigation; writing – review and editing. **Peter Macheroux:** Conceptualization; funding acquisition; methodology; writing – review and editing; project administration; supervision; resources; validation. **Aleksandar Bijelic:** Conceptualization; investigation; data curation; formal analysis; methodology; supervision; validation; visualization; writing – original draft; writing – review and editing.

## FUNDING INFORMATION

This research project was funded by the Austrian Science Fund (FWF) (grant 10.55776/DOC46). For the purpose of open access, the author has applied a CC BY public copyright license to any Author Accepted Manuscript version arising from this submission.

## CONFLICT OF INTEREST STATEMENT

The authors declare no conflict of interest.

## Supporting information


**Figure S1.** Results of the NAD(P)H acceptance assays for some selected variants. Results are shown for (A) R283E, (B) R343N, (C) Y364P, (D) R366A, (E) L6‐8aa, (F) L6‐9aa, (G) L6‐9aa/R283D, and (H) L6‐*Achr*OYE4. The spectrum of each of the 15 cycles is shown (see legend). The gray dotted spectra represent those before adding the enzyme.
**Figure S2.** Stopped‐flow pre‐steady‐state kinetics of *Sl*OPR3 variants. (A) Representative figure for the time‐dependent spectral changes during FMN reduction by NAD(P)H. (B–H) Concentration dependence of FMN reduction in different *Sl*OPR3 variants (see labels) with NADPH (blue) vs. NADH (red). All measurements were performed in triplicate. Standard deviations are indicated as error bars. FMN reduction in swap variant L6‐*Achr*OYE4 (G and H) was biphasic.
**Figure S3.** Crystal structure of the R366A–NADPH_4_ complex. The overall structure is shown on the left, with the bound NADPH_4_ molecule in the background (purple sticks). The inset shows a zoomed view of the active site of R366A (gray cartoon and sticks) superimposed with the active site of the wild type (green cartoon sticks, PDB: 8QMX). All residues superimpose well, except for the substituted position 366.
**Figure S4.** Crystal structure of the R283D–NADPH_4_ complex. The overall tetrameric structure is shown in the middle, representing a dimer of dimers. Protomers A and B form the previously reported ‘semi‐self‐inhibitory’ dimer [REF]. Protomers C and D form a crystallographic dimer, with protomer D being a disordered, cosubstrate‐free protomer, and protomer C being NADPH_4_‐bound. Insets show a zoomed and detailed view of the cosubstrate–enzyme interactions.
**Figure S5.** Ancestral sequence reconstruction based on 98 OYE sequences. OYE classes are indicated (I–VI) and color‐coded. OYEs not color‐coded could not be assigned to an OYE class. Colored circles indicate how many of the four OPR3‐like motif residues are present in the enzymes and ancestors (nodes); see legend for color code. The tree is a maximum‐likelihood consensus tree based on 1000 bootstrapped replicates. Posterior values for relevant nodes are summarized in Table S8.
**Table S1.** Curated list of NADPH‐preferring ERs.
**Table S2.** Steady‐state kinetic parameters for *Sl*OPR3 wild‐type and variants with NADPH (cosubstrate) and 2‐methylmaleimide (substrate).
**Table S3.** Data collection and refinement statistics for R283D in complex with NAD(P)H_4_.
**Table S4.** Data collection and refinement statistics for R283E in complex with NAD(P)H_4_.
**Table S5.** Data collection and refinement statistics for L6‐8aa with and without NAD(P)H_4_.
**Table S6.** Data collection and refinement statistics for L6‐9aa with and without NAD(P)H_4_.
**Table S7.** Data collection and refinement statistics for L6‐*Achr*OYE4 + NAD(P)H_4_.
**Table S8.** Data collection and refinement statistics for R366A.
**Table S9.** Node‐wise posteriors and motif calls for the ancestral sequence reconstruction.
**Table S10.** Primer sequences for all single and double amino acid substation variants of *Sl*OPR3.
**Table S11.** Crystallization conditions for all *Sl*OPR3 variants.

## Data Availability

The data supporting the findings of this study are available from the wwPDB under the accession codes 8QNA, 8QNE, 8QNM, 8QNK, 8QNP, 8QNW, 8QNX, 8QNY, 8QO6, 8QO7, 8QO8, 9FCN, and 9FCP.
