# Supplementary material for: Structural and evolutionary dissection of NADPH‐binding motifs in NADPH‐preferring ene‐reductases
Source: Protein Sci. 2026 Mar 18;35(4):e70521. doi: 10.1002/pro.70521 (PMC13140739; doi:10.1002/pro.70521)
Supplement: Supplementary file 1 — Figure S1. Results of the NAD(P)H acceptance assays for some selected variants. Results are shown for (A) R283E, (B) R343N, (C) Y364P, (D) R366A, (E) L6‐8aa, (F) L6‐9aa, (G) L6‐9aa/R283D, and (H) L6‐AchrOYE4. The spectrum of each of the 15 cycles is shown (see legend). The gray dotted spectra represent those before adding the enzyme. Figure S2. Stopped‐flow pre‐steady‐state kinetics of SlOPR3 variants. (A) Representative figure for the time‐dependent spectral changes during FMN reduction by NAD(P)H. (B–H) Concentration dependence of FMN reduction in different SlOPR3 variants (see labels) with NADPH (blue) vs. NADH (red). All measurements were performed in triplicate. Standard deviations are indicated as error bars. FMN reduction in swap variant L6‐AchrOYE4 (G and H) was biphasic. Figure S3. Crystal structure of the R366A–NADPH4 complex. The overall structure is shown on the left, with the bound NADPH4 molecule in the background (purple sticks). The inset shows a zoomed view of the active site of R366A (gray cartoon and sticks) superimposed with the active site of the wild type (green cartoon sticks, PDB: 8QMX). All residues superimpose well, except for the substituted position 366. Figure S4. Crystal structure of the R283D–NADPH4 complex. The overall tetrameric structure is shown in the middle, representing a dimer of dimers. Protomers A and B form the previously reported ‘semi‐self‐inhibitory’ dimer [REF]. Protomers C and D form a crystallographic dimer, with protomer D being a disordered, cosubstrate‐free protomer, and protomer C being NADPH4‐bound. Insets show a zoomed and detailed view of the cosubstrate–enzyme interactions. Figure S5. Ancestral sequence reconstruction based on 98 OYE sequences. OYE classes are indicated (I–VI) and color‐coded. OYEs not color‐coded could not be assigned to an OYE class. Colored circles indicate how many of the four OPR3‐like motif residues are present in the enzymes and ancestors (nodes); see legend for color code. The tree is a [file PRO-35-e70521-s001.docx]

**Structural and evolutionary dissection of NADPH binding motifs in NADPH-preferring ene-reductases**

Bianca Kerschbaumer^1^, Eva M. Frießer^1^, Silvia Wallner^1^, Gustav Overdorfer^1,3^, Michael Friess^2^, Rolf Breinbauer^2^, Peter Macheroux^1^*, Aleksandar Bijelic^1^*

^1^Institute of Biochemistry, Graz University of Technology, Graz, Austria

^2^Institute of Organic Chemistry, Graz University of Technology, Graz, Austria

^3^BioTechMed-Graz, Graz, Austria

**Supporting Figures**


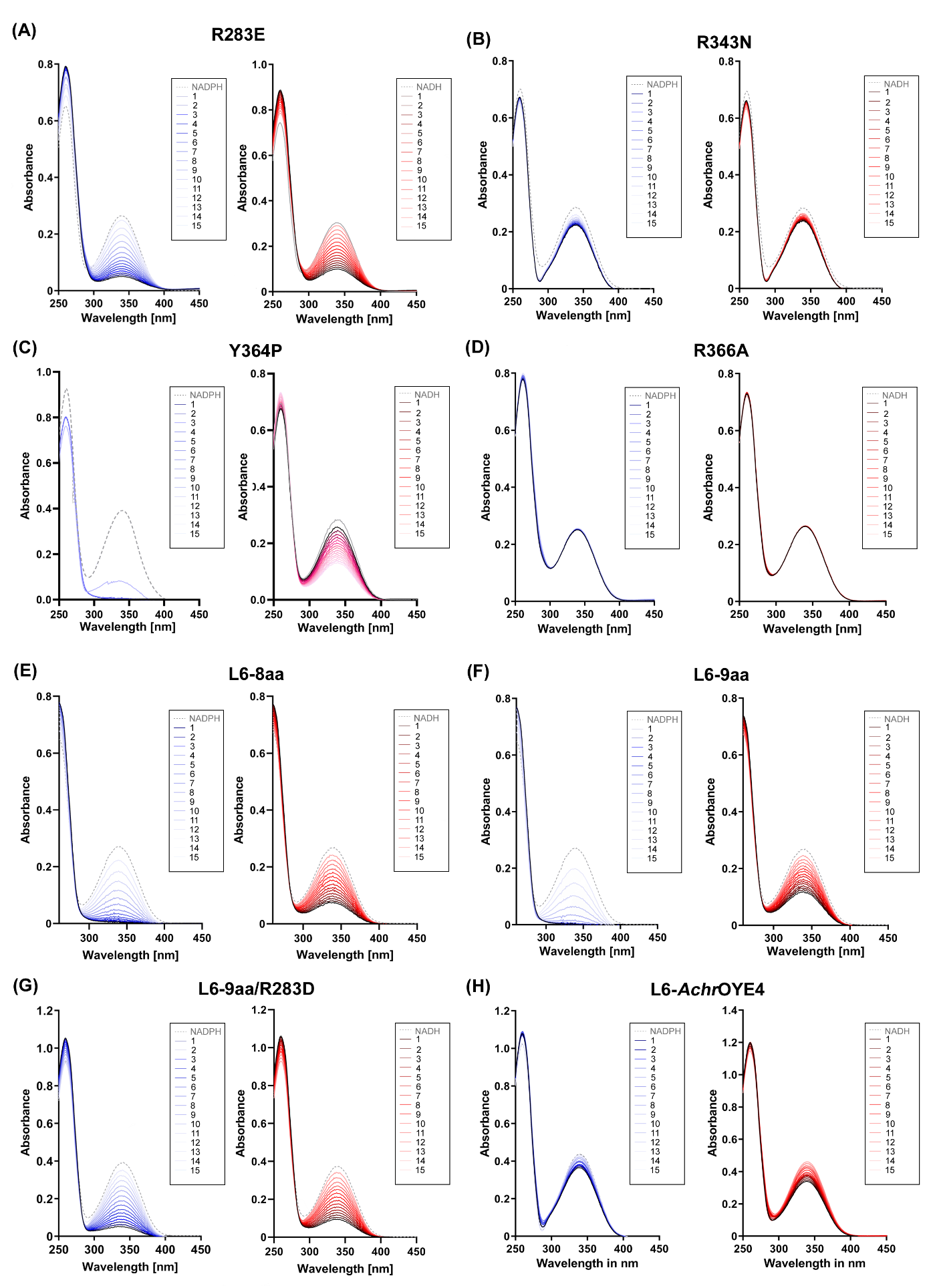


**Figure S1.** Results of the NAD(P)H acceptance assays for some selected variants. Results are shown for (A) R283E, (B) R343N, (C) Y364P, (D) R366A, (E) L6-8aa, (F) L6-9aa, (G) L6-9aa/R283D, and (H) L6-*Achr*OYE4. The spectrum of each of the 15 cycles is shown (see legend). The grey dotted spectra represent those before adding the enzyme.

**
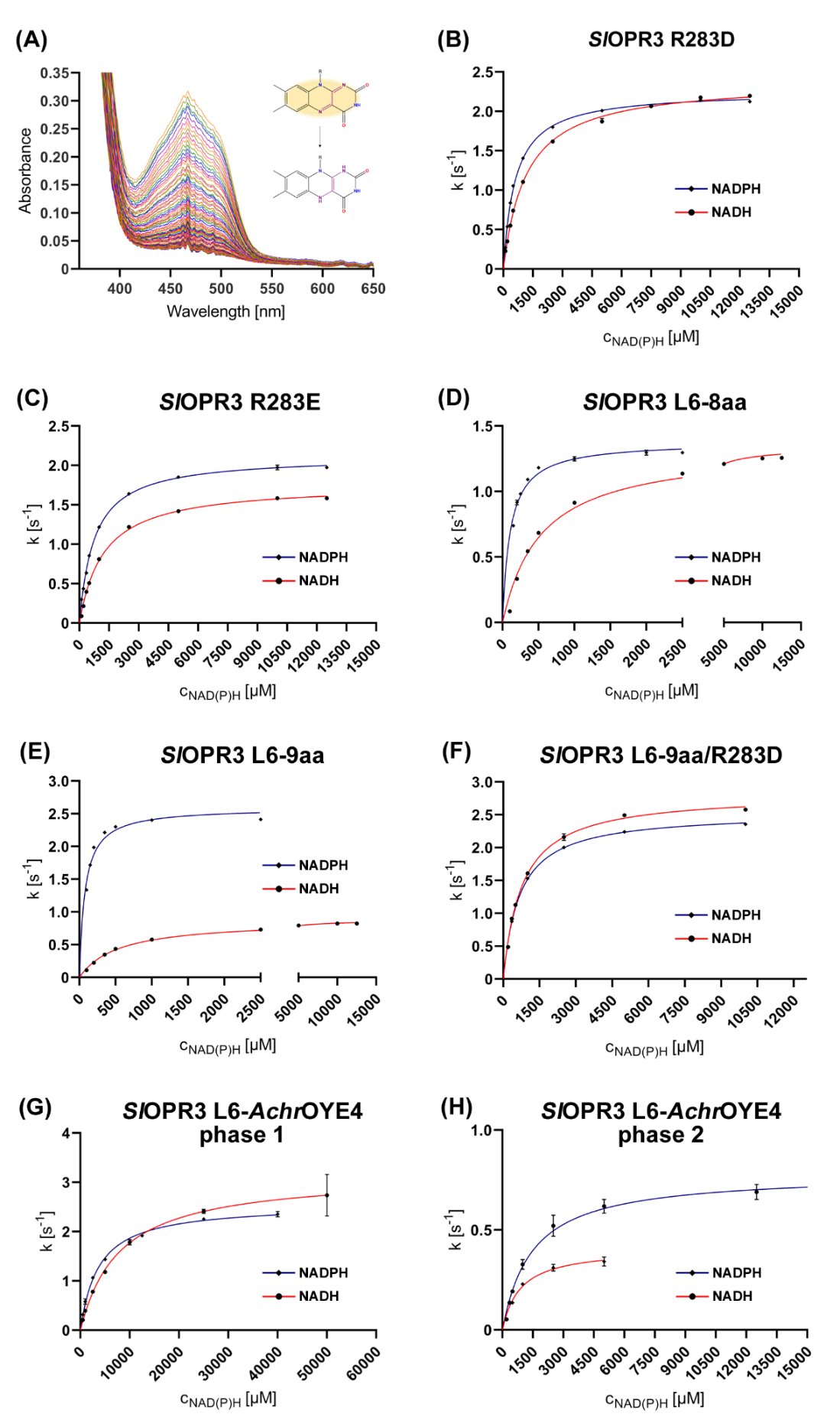
**

**Figure S2.** Stopped-flow pre-steady-state kinetics of *Sl*OPR3 variants. (A) Representative figure for the time-dependent spectral changes during FMN reduction by NAD(P)H. (B–H) Concentration dependence of FMN reduction in different *Sl*OPR3 variants (see labels) with NADPH (blue) vs. NADH (red). All measurements were performed in triplicate. Standard deviations are indicated as error bars. FMN reduction in swap variant L6-*Achr*OYE4 (G and H) was biphasic.

**
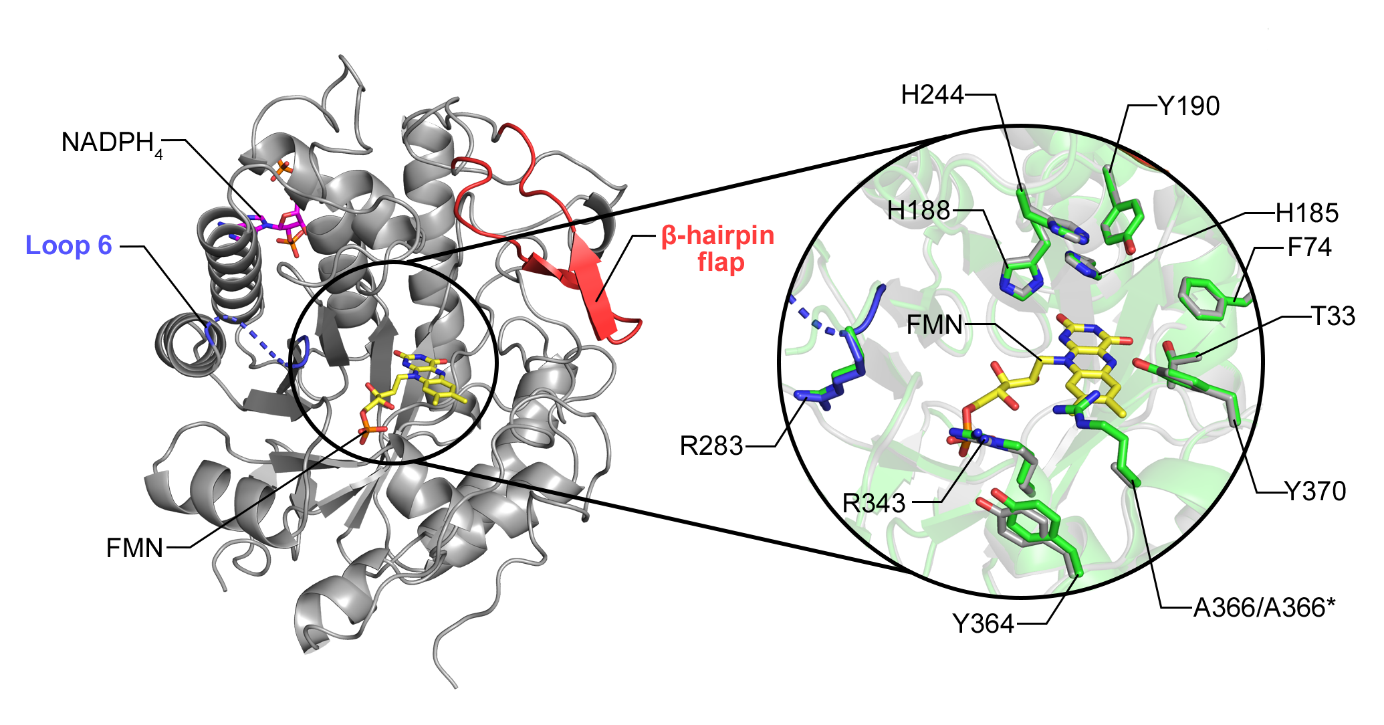
Figure S3.** Crystal structure of the R366A–NADPH_4_ complex. The overall structure is shown on the left, with the bound NADPH_4_ molecule in the background (purple sticks). The inset shows a zoomed view of the active site of R366A (grey cartoon and sticks) superimposed with the active site of the wild type (green cartoon sticks, PDB: 8QMX). All residues superimpose well, except for the substituted position 366.

The crystal structure of R366A–NADPH_4_ is identical to that of the wild type (RMSD_Cα_ = 0.17 Å). L6 lacks 11 amino acids (V285–G296). The NADPH_4_ molecule bound at the enzyme's surface and is situated on a crystallographic twofold axis, indicating that this binding site is a crystallographic artifact. Thus, the crystal structure confirms that R366 is indispensable for NADPH binding, as the variant failed to bind the cosubstrate despite high cosubstrate concentrations, corroborating our results.


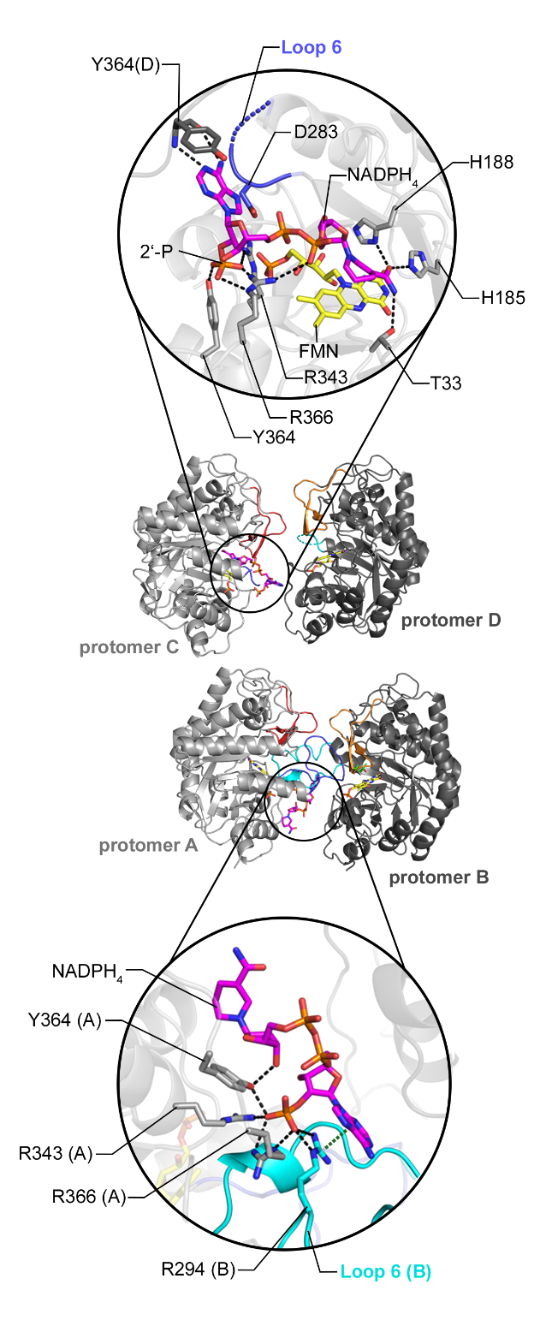


**Figure S4.** Crystal structure of the R283D–NADPH_4_ complex. The overall tetrameric structure is shown in the middle, representing a dimer of dimers. Protomers A and B form the previously reported 'semi-self-inhibitory' dimer [REF]. Protomers C and D form a crystallographic dimer, with protomer D being a disordered, cosubstrate-free protomer, and protomer C being NADPH_4_-bound. Insets show a zoomed and detailed view of the cosubstrate–enzyme interactions.

The NADPH_4_ molecule found in the semi-self-inhibitory dimer binds peripherally to the active site of chain A, which is blocked by L6 of chain B. In this position, the 2’-P group of NADPH_4_ exhibits strong ion-ion interactions with R343 and R366 of chain A and R294 of chain B. Additionally, it H-bonds with Y364 of chain A. The adenine ring of NADPH_4_ is involved in cation-π stacking interactions with R294 of chain B. It is unclear whether this NADPH_4_ binding mode represents a pre-binding state or is a crystallographic artifact. Chain D is cosubstrate-free and highly disordered. The cosubstrate-bound chain C is discussed in the main text.


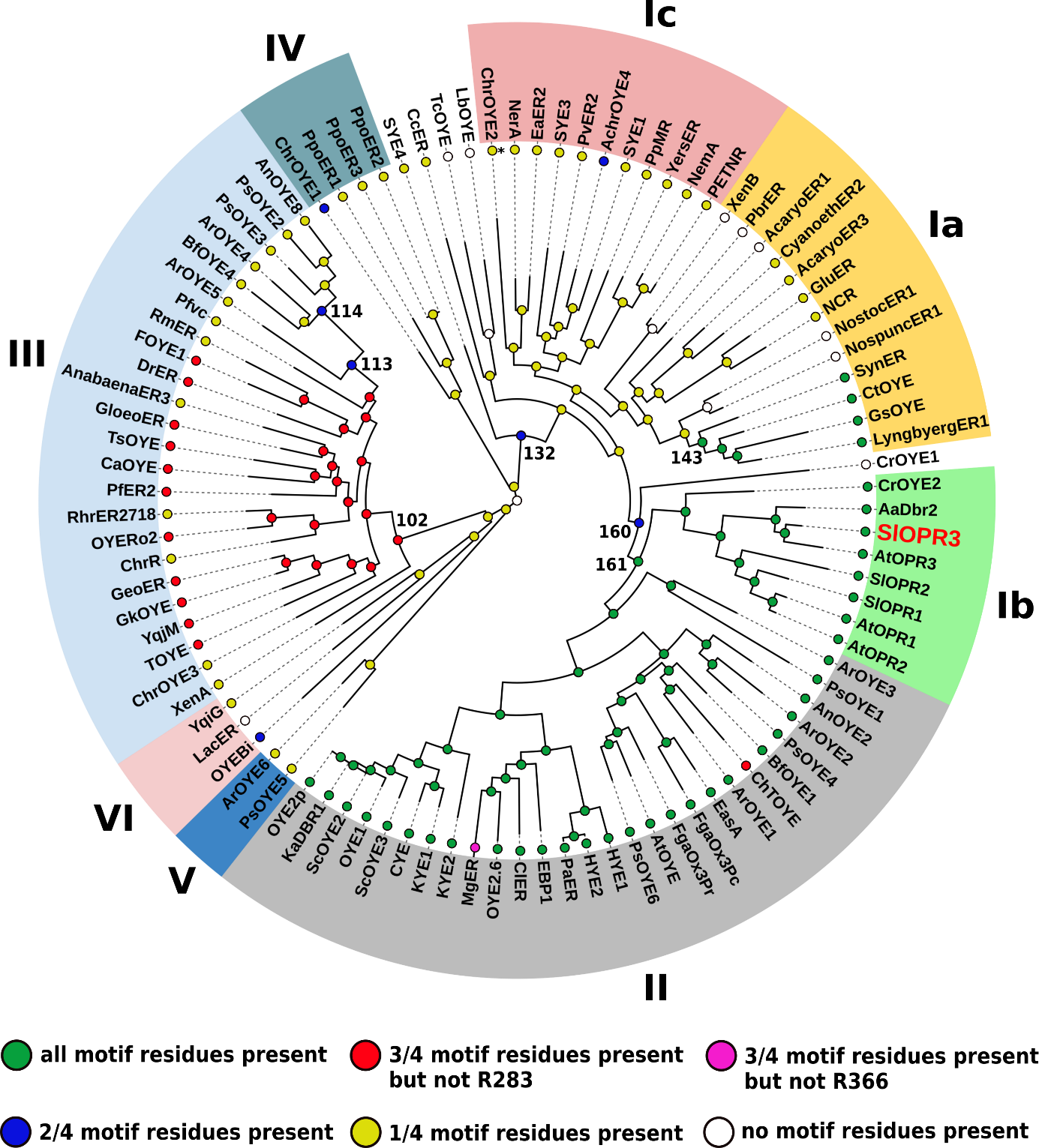


**Figure S5. Ancestral sequence reconstruction based on 98 OYE sequences.** OYE classes are indicated (I–VI) and color-coded. OYEs not color-coded could not be assigned to an OYE class. Colored circles indicate how many of the four OPR3-like motif residues are present in the enzymes and ancestors (nodes); see legend for color code. The tree is a maximum-likelihood consensus tree based on 1000 bootstrapped replicates. Posterior values for relevant nodes are summarized in Table S8.

To trace the evolutionary emergence and distribution of the OPR3‑like NADPH‑binding motif, we performed ancestral sequence reconstruction on 98 OYE sequences (Figure S5). The analysis revealed a stepwise assembly of the four residues (R283/R343/Y364/R366) and composite motifs (2’-P site/adenine clamp). For interpretation, we used per-site posterior thresholds present (P ≥ 0.70), ambiguous (0.50 ≤ P < 0.70), and absent (P < 0.50) at these four sites; node-wise values are summarized below and in Table S8.

At the global root, none of the residues is confidently inferred (P < 0.3 for all residues), and we therefore did not interpret root states. R343 emerged earliest, consistent with its second role in FMN cofactor binding. Y364 appeared early but was frequently lost and regained, underscoring its dispensability (first and second emergence at nodes 132 and 160, with P ≥ 0.7). The full motif appears at node 161 (P = 0.98/0.98/0.99/0.99 for R283/R343/Y364/R366) and is largely retained in descent OYE classes Ib (plants) and II (fungi) (see nodes N161 in Table S8), consistent with our motif-conservation data. In some clades, R366 occurs earlier than R283, but along the *Sl*OPR3 path, these coincide at node 161. The immediate predecessor N160 already supports R343/Y364 (P = 1.0 and 0.93, respectively) while R366 (P = 0.53) is ambiguous and R283 absent (P = 0.21), consistent with an anchor-first, clamp-later progression.

A second, independent gain of the full motif occurs in a cyanobacterial class‑I lineage at node 143 (P = 0.98/1.0/0.99/0.98 for R283/R343/Y364/R366), indicating convergent evolution of OPR3‑like NADPH recognition.

Outside these clades, class‑I lineages lacking R283/R366 likely rely on alternative, often NADH‑favoring binding modes, such as those exhibited by morphinone reductase. In class III, the most recent common ancestor (N102) supports the 2’-P anchor (P = 1.00/ 0.92/0.89 for R343/Y364/R366) but lacks the clamp (P = 0.00 for R283), consistent with clamp-free, often dimerization-assisted strategies; within this branch, nodes such as N113 (P = 0.00/1.0/0.50/0.97 for R283/R343/Y364/R366) and N114 (0.00/1.00/0.00/0.00 for R283/R343/Y364/R366) illustrate turnover at the anchor positions. These patterns correlate with the appearance of the arginine finger and tryptophan finger motifs, which are characteristic of thermophilic-like OYEs, such as YqjM and XenA, respectively.

Overall, the results support a model in which NADPH binding is clade‑ and class‑specific. While OPR3‑like motifs arose convergently in plants/fungi and some cyanobacteria, other lineages evolved distinct NADPH‑binding strategies, emphasizing both the plasticity and the modularity of cosubstrate recognition across the OYE family.

**Supporting Tables**

**Table S1.** Curated list of NADPH-preferring ERs.

| **ER** | **kingdom** | **OYE**  **class** | **NADPH**  **preference^[a]^** | **R283^[b]^** | **R343^[b]^** | **R364^[b]^** | **R366^[b]^** | **2’-P**  **site** | **adenine**  **clamp** | **Ref.** |
| --- | --- | --- | --- | --- | --- | --- | --- | --- | --- | --- |
| *Sl*OPR3 | plant | I | 857×^[c]^ | R283 | R343 | Y364 | R366 | y | y | (Kerschbaumer et al., 2024) |
| AaDbr2 | plant | I | 15×^[d]^ | R278 | R340 | Y361 | R363 | y | y | (Zhang et al., 2008) |
| *Cr*OYE2 | plant | I | ~2×^[e]^ | R334 | R358 | Y406 | R408 | y | y | (Böhmer et al., 2023) |
| *Gs*OYE | plant | I | 9×^[d]^ | R286 | R343 | Y360 | R362 | y | y | (Robescu et al., 2020) |
| *Ct*OYE | bacteria | I | 32×^[d]^ | R270 | R324 | Y345 | R347 | y | y | (Robescu et al., 2020) |
| *Glu*ER | bacteria | I | 8×^[e]^ | G264 | R316 | D337 | M339 | n | n | (Richter et al., 2011) |
| NemA | bacteria | I | 2×^[e]^ | D275 | R325 | Q346 | A348 | n | n | (Williams et al., 2004) |
| *Pbr*-ER | bacteria | I | 17×^[f]^ | -^[i]^ | V309 | A330 | P332 | n | n | (Papadopoulou et al., 2022) |
| PETNR | bacteria | I | 171×^[c]^ | D274 | R324 | Q345 | P347 | n | n | (Iorgu et al., 2019) |
| *Ye*rs-ER | bacteria | I | 23×^[e]^ | W279 | R328 | P350 | G352 | n | n | (Chaparro‐Riggers et al., 2007) |
| AcaryoER1 | cyanobacteria | I | ~3×^[e]^ | L268 | V319 | P340 | P342 | n | n | (Mähler et al., 2019) |
| AcaryoER3 | cyanobacteria | I | ~4×^[e]^ | L273 | R324 | A346 | Q348 | n | n | (Fu et al., 2013) |
| CyanothER2 | cyanobacteria | I | ~5×^[e]^ | E274 | R324 | A346 | M248 | n | n | (Fu et al., 2013) |
| LyngbyaER1 | cyanobacteria | I | ~39×^[e]^ | R269 | R323 | Y344 | R346 | y | y | (Fu et al., 2013) |
| NospuncER1 | cyanobacteria | I | ~5×^[e]^ | T269 | T320 | A341 | Q343 | n | n | (Fu et al., 2013) |
| NostocER1 | cyanobacteria | I | ~13×^[e]^ | I306 | T357 | A378 | P380 | n | n | (Mähler et al., 2019) |
| *Syn*ER | cyanobacteria | I | ~22×^[e]^ | R270 | R323 | Y344 | R346 | y | y | (Fu et al., 2012) |
| *An*OYE2 | fungi | II | dep.^[e]^ | R262 | R318 | Y339 | R341 | y | y | (Robescu et al., 2022) |
| *At*OYE | fungi | II | 2×^[e]^ | R260 | R317 | Y338 | R340 | y | y | (Damada and Fraaije, 2024) |
| *Bf*OYE1 | fungi | II | dep.^[e]^ | R262 | R318 | Y339 | R341 | y | y | (Robescu et al., 2022) |
| *Cht*OYE | fungi | II | 6×^[e]^ | G266 | K321 | Y341 | R343 | y | n | (Damada and Fraaije, 2024) |
| *Cl*ER | fungi | II | 106×^[f]^ | R285 | R343 | Y364 | R366 | y | y | (Ni et al., 2014) |
| KYE1 | fungi | II | 14×^[e]^ | R295 | R351 | Y372 | R374 | y | y | (Chaparro‐Riggers et al., 2007) |
| KYE2 | fungi | II | 7×^[e]^ | R226 | R282 | Y302 | R304 | y | y | (Li et al., 2018) |
| *Lt*OYE | fungi | II | 3×^[e]^ | R292 | R350 | Y371 | R373 | y | y | (Damada and Fraaije, 2024) |
| *Mg*ER | fungi | II | 84×^[e]^ | R279 | S333 | Y354 | R356 | y | y | (Zhang et al., 2016) |
| OYE1 | fungi | II | 10×^[e]^ | R291 | R348 | Y369 | R371 | y | y | (Williams et al., 2004) |
| *Op*OYE | fungi | II | 2×^[e]^ | R346 | R402 | Y423 | Y425 | y | y | (Damada and Fraaije, 2024) |
| *Pa*ER | fungi | II | 32×^[e]^ | R285 | R342 | Y363 | R365 | y | y | (Zhang et al., 2022) |
| *Ps*OYE1 | fungi | II | 2×^[e]^ | R299 | R362 | Y383 | R385 | y | y | (Damada et al., 2025) |
| *Ps*OYE4 | fungi | II | 2×^[e]^ | R261 | R340 | Y338 | R340 | y | y | (Damada et al., 2025) |
| *Ps*OYE6 | fungi | II | 2×^[e]^ | R262 | R342 | Y340 | R342 | y | y | (Damada et al., 2025) |
| *Tt*OYE | fungi | II | 2×^[e]^ | R267 | R319 | Y340 | R342 | y | y | (Damada and Fraaije, 2024) |
| *Dr*ER | bacteria | III | ~20×^[g]^ | G281 | R339 | Y365* | R367* | y | n | (Litthauer et al., 2014) |
| FOYE-1 | bacteria | III | 27×^[d]^ | V276 | R324 | Y350* | R352* | y | n | (Scholtissek et al., 2017) |
| *Ts*OYE | bacteria | III | 32×^[c]^ | L271 | R319 | Y345* | R347* | y | n | (Knaus et al., 2016) |
| TOYE | bacteria | III | 60×^[c]^ | G251 | R307 | Y331* | R333* | y | n | (Knaus et al., 2016) |
| YqjM | bacteria | III | 46×^[c]^ | G263 | R308 | Y334* | R336* | y | n | (Assil-Companioni et al., 2020) |
| *An*OYE8 | fungi | III | dep.^[e]^ | G322 | R369 | F399* | W397* | n | n | (Robescu et al., 2022) |
| *Bf*OYE4 | fungi | III | dep.^[e]^ | A335 | R402 | -^[i]^ | -^[i]^ | n | n | (Robescu et al., 2022) |
| *Ps*OYE3 | bacteria | III | 2×^[e]^ | G316 | R375 | I401* | W403* | n | n | (Damada et al., 2025) |
| *Rm*ER | bacteria | III | ~4×^[g]^ | G268 | R325 | Q346 | P348 | n | n | (Litthauer et al., 2014) |
| XenA | bacteria | III | 22×^[c]^ | W278 | R326 | A356* | W358* | n | n | (Knaus et al., 2016) |
| *Chr*OYE1 | bacteria | IV | ~4×^[g]^ | G268 | R325 | Q346 | P348 | n | n | (Pei et al., 2016) |
| *Ar*OYE6 | fungi | V | dep.^[d]^ | -^[i]^ | R348 | -^[i]^ | -^[i]^ | n | n | (Singh et al., 2022) |
| *Ps*OYE5 | fungi | V | 3×^[e]^ | F294 | R351 | -^[i]^ | -^[i]^ | n | n | (Damada et al., 2025) |
| YqiG | bacteria | VI | dep.^[f]^ | D263 | R319 | P369 | W367 | n | n | (Sheng et al., 2016) |
| *Cr*OYE1 | plant | n.d. | ~10×^[c]^ | N277 | V335 | F361 | A363 | n | n | (Böhmer et al., 2023) |
| SYE4 | bacteria | n.d. | 15×^[g]^ | D274 | R325 | Y346 | D348 | n | n | (Brigé et al., 2006) |
| *Lb*OYE | protista | n.d. | 3×^[h]^ | D278 | T325 | P346 | P348 | n | n | (Libardi et al., 2024) |
| *Tc*OYE | protista | n.d. | 3×^[h]^ | D289 | A337 | P358 | P360 | n | n | (Libardi et al., 2024) |

^[a]^ NADPH preference is shown as ×-fold over NADH (defined as NADPH activity/NADH activity as reported in the respective paper). ^[b]^ Residues at the corresponding positions to R283, R343, Y364, and R366 in OPR3 are shown. ^[c]^ Preference based on pre-steady state kinetics ([*k*_red_/K_d_]_NADPH_/[*k*_red_/K_d_]_NADH_). ^[d]^ Preference based on steady state kinetics ([*k*_cat_/K_m_]_NADPH_/[*k*_cat_/K_m_]_NADH_). ^[e]^ Preference based on specific activity ([U/mg]_NADPH_/[U/mg]_NADH_). ^[f]^ Preference based on monitoring NAD(P)H oxidation (NADPH oxidation/NADH oxidation). ^[g]^ Preference based on biotransformation (product amount using NADPH/product amount using NADH). ^[h]^ Preference based on initial enzyme rates (*k*_cat_NADPH*/k*_cat_NADH). ^[i]^ No spatially overlapping residue was found due to substantial structural differences. Green-shaded values indicate the presence of the motif residue and/or residue. Asterisks (*) indicate that the residue comes from an adjacent protomer, as the binding motif is built by a dimer. Tildes (~) indicate that the values were roughly estimated from graphs, as no values were reported in the respective paper. y, yes (present); n, no (absent); dep., dependent (i.e., no activity with NADH was measured); n.d., not defined (OYE could not be unambiguously assigned to a class).

**Table S2.** Steady-state kinetic parameters for *Sl*OPR3 wild-type and variants with NADPH (cosubstrate) and 2-methylmaleimide (substrate).

| **Enzyme** | **K_M_ [µM]** | **v_max_ [µM min^-1^]** | ***k*_cat_ [s^-1^]** |
| --- | --- | --- | --- |
| Wild type | 1511.3 ± 57.7 | 257.9 ± 1.8 | 17.19 |
| L6-8aa | 102.5 ± 10.8 | 23.1 ± 0.7 | 1.54 |
| L6-9aa | 175.3 ± 12.2 | 22.2 ± 1.3 | 1.48 |
| L6-9aa/R283D | 107.6 ± 15.7 | 31.2 ± 1.0 | 2.08 |
| R283D | 49.8 ± 5.4 | 6.6 ± 1.0 | 0.44 |
| R283E | 75.1 ± 8.2 | 7.5 ± 0.8 | 0.50 |

**Table S3.** Data collection and refinement statistics for R283D in complex with NAD(P)H_4_.

|  | **OPR3 R283D + NADPH_4_** | **OPR3 R283D + NADH_4_** |
| --- | --- | --- |
| **Data collection** |  |  |
| Diffraction source | ESRF, ID23-1 | DESY, P11 |
| Wavelength (Å) | 0.89 | 1.03 |
| Temperature (K) | 100 | 100 |
| Detector | Eiger2 X16M | Eiger2 X16M |
| Space group | P 1 2_1_ 1 | P 1 2_1_ 1 |
| *a*, *b*, *c* (Å) | 80.58, 90.48, 114.63 | 49.56, 92.85, 89.77 |
| *α*, *β*, *γ* (°) | 90, 110.02, 90 | 90, 97.86, 90 |
| No. of observed reflections | 460187 (12616) | 216284 (27969) |
| No. of unique reflections | 114487 (3915) | 32292 (4020) |
| Multiplicity | 4.0 (3.2) | 6.7 (6.9) |
| Completeness (%) | 95.49 (98.37) | 98.49 (98.72) |
| R_pim_ (%) | 5.38 (91.88) | 38.05 (172.1) |
| Mean *I*/σ(*I*) | 8.56 (0.85) | 3.52 (0.92) |
| CC_1/2_ | 0.993 (0.417) | 0.923 (0.197) |
|  |  |  |
| **Refinement** |  |  |
| Resolution range (Å) | 35.9 - 1.91 (1.93 - 1.91) | 45.71 - 2.37 (2.47 - 2.37) |
| R_work_/R_free_ (%) | 19.56/22.24 | 23.34/27.56 |
| RMS (bonds) (Å) | 0.009 | 0.008 |
| RMS (angles) (°) | 0.91 | 0.81 |
| Average *B*-factor (Å^2^) | 33.50 | 34.58 |
|  |  |  |
| **Ramachandran plot** |  |  |
| Ramachandran favored (%) | 96.56 | 95.47 |
| Ramachandran allowed (%) | 3.44 | 4.53 |
| Ramachandran outlier (%) | 0.00 | 0.00 |
|  |  |  |
| **PDB ID** | 8QNK | 8QO6 |

**Table S4.** Data collection and refinement statistics for R283E in complex with NAD(P)H_4_.

|  | **OPR3 R283E + NADPH_4_** | **OPR3 R283E + NADH_4_** |
| --- | --- | --- |
| **Data collection** |  |  |
| Diffraction source | DESY, P11 | DESY, P11 |
| Wavelength (Å) | 1.03 | 1.03 |
| Temperature (K) | 100 | 100 |
| Detector | Eiger2 X 16 M | Eiger2 X 16 M |
| Space group | P 2_1_ 2_1_ 2 | P 1 2_1_ 1 |
| *a*, *b*, *c* (Å) | 88.4, 90.27, 49.48 | 49.47, 93.32, 89.40 |
| *α*, *β*, *γ* (°) | 90, 90, 90 | 90, 97.73, 90 |
| No. of observed reflections | 1894205 (37199) | 905053 (44830) |
| No. of unique reflections | 78424 (2628) | 132566 (8502) |
| Multiplicity | 24.2 (14.1) | 6.8 (5.2) |
| Completeness (%) | 99.66 (94.74) | 97.17 (81.15) |
| R_pim_ (%) | 1.83 (23.13) | 3.87 (78.23) |
| Mean *I*/σ(*I*) | 31.68 (5.96) | 9.88 (0.90) |
| CC_1/2_ | 0.999 (0.812) | 0.998 (0.62) |
|  |  |  |
| **Refinement** |  |  |
| Resolution range (Å) | 45.13 - 1.4 (1.42 - 1.4) | 46.66 - 1.47 (1.51 - 1.47) |
| R_work_/R_free_ (%) | 15.14/17.07 | 18.74/20.48 |
| RMS (bonds) (Å) | 0.017 | 0.010 |
| RMS (angles) (°) | 1.54 | 1.09 |
| Average *B*-factor (Å^2^) | 17.51 | 28.35 |
|  |  |  |
| **Ramachandran plot** |  |  |
| Ramachandran favored (%) | 97.15 | 97.30 |
| Ramachandran allowed (%) | 2.85 | 2.70 |
| Ramachandran outlier (%) | 0.00 | 0.00 |
|  |  |  |
| **PDB ID** | 8QO7 | 8QO8 |

**Table S5.** Data collection and refinement statistics for L6-8aa with and without NAD(P)H_4_.

|  | **L6-8aa** | **L6-8aa + NADPH_4_** | **L6-8aa + NADH_4_** |
| --- | --- | --- | --- |
| **Data collection** |  |  |  |
| Diffraction source | DESY, P11 | ESRF ID30A-1 | DESY, P11 |
| Wavelength (Å) | 1.03 | 0.97 | 1.03 |
| Temperature (K) | 100 | 100 | 100 |
| Detector | Eiger2 X 16 M | PILATUS3 6M | Eiger2 X 16 M |
| Space group | P 2_1_ 2_1_ 2 | P 1 2_1_ 1 | P 2_1_ 2_1_ 2 |
| *a*, *b*, *c* (Å) | 88.03, 89.76, 49.57 | 49.74, 91.73, 89.77 | 88.01, 89.62, 49.40 |
| *α*, *β*, *γ* (°) | 90, 90, 90 | 90, 98.99, 90 | 90, 90, 90 |
| No. of observed reflections | 523244 (10512) | 632860 (10981) | 1752169 (41437) |
| No. of unique reflections | 55232 (1999) | 83145 (1445) | 65357 (1802) |
| Multiplicity | 9.5 (5.3) | 7.6 (7.4) | 26.8 (23.0) |
| Completeness (%) | 95.61 (70.44) | 87.00 (45.44) | 93.36 (60.11) |
| R_pim_ (%) | 3.70 (77.00) | 6.92 (74.79) | 2.07 (22.92) |
| Mean *I*/σ(*I*) | 11.85 (0.89) | 8.50 (0.68) | 23.80 (3.29) |
| CC_1/2_ | 0.999 (0.444) | 0.995 (0.483) | 0.999 (0.862) |
|  |  |  |  |
| **Refinement** |  |  |  |
| Resolution range (Å) | 44.01 - 1.55 (1.58 - 1.55) | 49.13 - 1.65 (1.67 - 1.65) | 44.01 - 1.45 (1.47 - 1.45) |
| R_work_/R_free_ (%) | 15.92/19.18 | 18.21/21.68 | 14.72/17.23 |
| RMS (bonds) (Å) | 0.010 | 0.012 | 0.015 |
| RMS (angles) (°) | 1.05 | 1.21 | 1.49 |
| Average *B*-factor (Å^2^) | 23.21 | 21.97 | 18.39 |
|  |  |  |  |
| **Ramachandran plot** |  |  |  |
| Ramachandran favored (%) | 97.51 | 97.08 | 97.50 |
| Ramachandran allowed (%) | 2.49 | 2.92 | 2.50 |
| Ramachandran outlier (%) | 0.00 | 0.00 | 0.00 |
|  |  |  |  |
| **PDB ID** | 8QNW | 8QNY | 8QNX |

**Table S6.** Data collection and refinement statistics for L6-9aa with and without NAD(P)H_4_.

|  | **L6-9aa** | **L6-9aa + NADPH_4_** | **L6-9aa + NADH_4_** |
| --- | --- | --- | --- |
| **Data collection** |  |  |  |
| Diffraction source | DESY, P11 | DESY, P11 | DESY, P11 |
| Wavelength (Å) | 1.03 | 1.03 | 1.03 |
| Temperature (K) | 100 | 100 | 100 |
| Detector | Eiger2 X 16 M | Eiger2 X 16 M | Eiger2 X 16 M |
| Space group | P 2_1_ 2_1_ 2 | P 1 2_1_ 1 | P 1 2_1_ 1 |
| *a*, *b*, *c* (Å) | 87.36, 90.10, 49.39 | 50.20, 89.95, 90.52 | 49.27, 91.79, 89.45 |
| *α*, *β*, *γ* (°) | 90, 90, 90 | 90, 100.38, 90 | 90, 98.48, 90 |
| No. of observed reflections | 907508 (13796) | 304667 (17293) | 365496 (19493) |
| No. of unique reflections | 75301 (2121) | 43647 (836) | 52480 (2756) |
| Multiplicity | 12.1 (6.5) | 6.5 (5.7) | 7.0 (7.1) |
| Completeness (%) | 97.17 (74.95) | 91.80 (26.62) | 98.60 (98.53) |
| R_pim_ (%) | 2.01 (76.57) | 27.63 (206.3) | 9.17 (86.63) |
| Mean *I*/σ(*I*) | 18.31 (0.94) | 1.27 (0.10) | 6.09 (0.85) |
| CC_1/2_ | 0.999 (0.38) | 0.618 (0.0917) | 0.994 (0.462) |
|  |  |  |  |
| **Refinement** |  |  |  |
| Resolution range (Å) | 43.68 - 1.4 (1.42 - 1.4) | 44.97 - 2.08 (2.12 - 2.08) | 45.9 - 2.0 (2.04 - 2.0) |
| R_work_/R_free_ (%) | 15.87/18.24 | 21.06/24.33 | 21.27/23.97 |
| RMS (bonds) (Å) | 0.022 | 0.002 | 0.017 |
| RMS (angles) (°) | 1.49 | 0.45 | 0.98 |
| Average *B*-factor (Å^2^) | 24.88 | 38.92 | 34.67 |
|  |  |  |  |
| **Ramachandran plot** |  |  |  |
| Ramachandran favored (%) | 97.50 | 96.95 | 97.34 |
| Ramachandran allowed (%) | 2.50 | 3.05 | 2.66 |
| Ramachandran outlier (%) | 0.00 | 0.00 | 0.00 |
|  |  |  |  |
| **PDB ID** | 8QNE | 8QNM | 8QNP |

**Table S7.** Data collection and refinement statistics for L6-*Achr*OYE4 + NAD(P)H_4_.

|  | **L6-*Ach*rOYE4 + NADPH_4_** | **L6-*Ach*rOYE4 + NADH_4_** |
| --- | --- | --- |
| **Data collection** |  |  |
| Diffraction source | ESRF ID30A-1 | ESRF ID30A-1 |
| Wavelength (Å) | 0.97 | 0.97 |
| Temperature (K) | 100 | 100 |
| Detector | Eiger2 X 16 M | Eiger2 X 16 M |
| Space group | P 1 2_1_ 1 | P 1 2_1_ 1 |
| *a*, *b*, *c* (Å) | 49.32, 93.99, 89.28 | 49.19, 92.96, 89.71 |
| *α*, *β*, *γ* (°) | 90, 97.60, 90 | 90, 97.93, 90 |
| No. of observed reflections | 356444 (23907) | 158587 (7532) |
| No. of unique reflections | 50345 (3357) | 52186 (2781) |
| Multiplicity | 7.1 (7.1) | 4.7 (3.9) |
| Completeness (%) | 98.19 (92.07) | 96.55 (97.85) |
| R_pim_ (%) | 0.09 (1.71) | 0.09 (0.52) |
| Mean *I*/σ(*I*) | 6.35 (0.25) | 5.55 (1.13) |
| CC_1/2_ | 0.99 (0.21) | 0.994 (0.462) |
|  |  |  |
| **Refinement** |  |  |
| Resolution range (Å) | 45.41 - 2-25 (2.31 - 2.25) | 45.44 - 2.0 (2.04 - 2.0) |
| R_work_/R_free_ (%) | 18.37/23.48 | 19.37/22.93 |
| RMS (bonds) (Å) | 0.003 | 0.002 |
| RMS (angles) (°) | 0.53 | 0.55 |
| Average *B*-factor (Å^2^) | 30.00 | 35.09 |
|  |  |  |
| **Ramachandran plot** |  |  |
| Ramachandran favored (%) | 96.63 | 97.15 |
| Ramachandran allowed (%) | 3.37 | 2.85 |
| Ramachandran outlier (%) | 0.00 | 0.00 |
|  |  |  |
| **PDB ID** | 9FCN | 9FCP |

**Table S8.** Data collection and refinement statistics for R366A.

|  | **R366A + NADPH^[a]^** |
| --- | --- |
| **Data collection** |  |
| Diffraction source | DESY, P11 |
| Wavelength (Å) | 1.03 |
| Temperature (K) | 100 |
| Detector | Eiger2 X 16 M |
| Space group | P 2_1_ 2_1_ 2 |
| *a*, *b*, *c* (Å) | 88.25, 90.33, 49.17 |
| *α*, *β*, *γ* (°) | 90, 90, 90 |
| No. of observed reflections | 554277 (17782) |
| No. of unique reflections | 62149 (2388) |
| Multiplicity | 8.9 (7.4) |
| Completeness (%) | 97.54 (83.55) |
| R_pim_ (%) | 6.41 (91.88) |
| Mean *I*/σ(*I*) | 9.41 (0.75) |
| CC_1/2_ | 0.997 (0.281) |
|  |  |
| **Refinement** |  |
| Resolution range (Å) | 49.17 - 1.5 (1.52 - 1.5) |
| R_work_/R_free_ (%) | 15.39/19.38 |
| RMS (bonds) (Å) | 0.008 |
| RMS (angles) (°) | 1.03 |
| Average *B*-factor (Å^2^) | 21.57 |
|  |  |
| **Ramachandran plot** |  |
| Ramachandran favored (%) | 97.21 |
| Ramachandran allowed (%) | 2.79 |
| Ramachandran outlier (%) | 0.00 |
|  |  |
| **PDB ID** | 8QNA |

^[a]^ Crystal was soaked with NADPH, but the cosubstrate only bound to the enzyme's surface as a result of a crystallographic artifact.

**Table S9.** Node-wise posteriors and motif calls for the ancestral sequence reconstruction.

| **node** | **lineage** | **P(R283)** | **P(R343)** | **P(Y364)** | **P(R366)** | **2’P-site** | **adenine**  **clamp** |
| --- | --- | --- | --- | --- | --- | --- | --- |
| N161 | OPR3-like (plant/fungal) | 0.98 | 1.00 | 0.99 | 0.99 | present | present |
| N160 | pre-OPR3  most recent ancestors | 0.21 | 0.98 | 0.93 | 0.53 | ambiguous | absent |
| N143 | Class-I/OPR3-like  (cyanobacterial) | 0.98 | 1.00 | 0.99 | 0.98 | present | present |
| N132 | early pre-OPR3  ancestors | 0.00 | 1.00 | 0.84 | 0.25 | absent | absent |
| N114 | Class-III  inner YqjM/XenA branch | 0.00 | 1.00 | 0.00 | 0.00 | absent | absent |
| N113 | Class-III  inner YqjM/XenA branch | 0.00 | 1.00 | 0.50 | 0.97 | ambiguous | absent |
| N102 | Class III  most recent ancestors | 0.00 | 1.00 | 0.92 | 0.89 | present | absent |

P = posterior probability. A residue and/or motif was classified as present, ambiguous, or absent when P was ≥ 0.70, 0.50 ≤ P < 0.70, or < 0.50, respectively.

**Table S10.** Primer sequences for all single and double amino acid substation variants of *Sl*OPR3.

| **Variant** | **Primer** | **Primer sequence 5’ 🡪 3’** |
| --- | --- | --- |
| R283D | Forward | CTTCATGTAACACAGCCAGACTACGTAGCATATGGGCA |
|  | Reverse | CTTCAGTTTGCCCATATGCTACGTAGTCTGGCTGTGTTAC |
| R283E | Forward | CTTCATGTAACACAGCCAGAATACGTAGCATATGGGCA |
|  | Reverse | CTTCAGTTTGCCCATATGCTACGTATTCTGGCTGTGTTAC |
| R343N | Forward | GCTGATCTCGTGTCATATGGTAATCTTTTCATCTCTAATCCT |
|  | Reverse | ACCAAATCAGGATTAGAGATGAAAAGATTACCATATGACACG |
| Y364P | Forward | TCTAAATAAGCCGAACAGGAAGACATTCTATAC |
|  | Reverse | GGTGCATTTAGCTTGATTC |
| R366A | Forward | CACCTCTAAATAAGTATAACGCAAAGACATTCTATACTC |
|  | Reverse | CTGGATCTTGAGTATAGAATGTCTTTGCGTTATAC |
| L6-*Achr*OYE4 | Forward | ATTGGGATGATGCGCCGGATATGCCGGAAGAGGAAGAGGCTCGTTTAATGAGGAC |
|  | Reverse | CATATCCGGCGCATCATCCCAATCCGCTTCCGCTGTTACATGAAGATAGGCAAGCTTGGAACC |
| L6-8aa | Forward | GATGGCCGCGGAAGTGAAGAGGAAGAGGCTCGTTTAATGAGGAC |
|  | Reverse | CGGCCATCGCCTGGTTGTGTTACATGAAGATAGGCAAGCTTGGAAC |
| L6-9aa | Forward | CCACGCAGCGATAAAGGCGGAAGTGAAGAGGAAGAGGCTCGTTTAATGAGGAC |
|  | Reverse | TATCGCTGCGTGGTTGTGTTACATGAAGATAGGCAAGCTTGGAACC |
| L6-9aa/R283D | * |  |
|  | * |  |

* primer pairs of the respective single amino acid substitution variants were used.

**Table S11.** Crystallization conditions for all *Sl*OPR3 variants.

| **variant** | **reservoir solution** | **drop setup** | **cosubstrate treatment** |
| --- | --- | --- | --- |
| R283D  NADPH_4_ | 100 mM Tris-HCl (7.5), 50 mM Na-tartrate, 10% PEG8000 | 1-2 µL protein^[a]^  1-2 µL reservoir  0.5 µL seeds (1:10) | soaked in cryo solution^[b]^ for 10 min |
| R283D  NADH_4_ | 100 mM Tris-HCl (7.5), 50 mM Na-tartrate, 10% PEG8000 + microseeds | 1-2 µL protein^[a]^  1-2 µL reservoir  0.5 µL seeds (1:10) | soaked in cryo solution^[b]^ for 40 min |
| R283E  NADPH_4_ | 100 mM MES/Tris (6.5), 10 mM (NH_4_)_2_SO_4_, 14% PEG8000 | 1-2 µL protein^[a]^  1-2 µL reservoir  0.5 µL seeds (1:10) | soaked in cryo solution^[b]^ for 40 min |
| R283E  NADH_4_ | 100 mM MES/Tris (6.5), 10 mM (NH_4_)_2_SO_4_, 14% PEG8000 | 1-2 µL protein^[a]^  1-2 µL reservoir  0.5 µL seeds (1:10) | soaked in cryo solution^[b]^ for 75 min |
| R366A | 100 mM Tris-HCl (7.5), 50 mM Na-tartrate, 18% PEG8000 | 1-2 µL protein^[a]^  1-2 µL reservoir | no treatment |
| L6-8aa | 100 mM MES/Tris (6.5), 50 mM (NH_4_)_2_SO_4_, 8% PEG8000 | 1-2 µL protein^[a]^  1-2 µL reservoir | no treatment |
| L6-8aa  NADPH_4_ | 100 mM MES/Tris (6.5), 50 mM (NH_4_)_2_SO_4_, 14% PEG8000 | 1-2 µL protein^[a]^  1-2 µL reservoir | soaked in reservoir solution^[c]^ for 5 min |
| L6-8aa  NADH_4_ | 100 mM MES/Tris (6.5), 50 mM (NH_4_)_2_SO_4_, 8% PEG8000 | 1-2 µL protein^[a]^  1-2 µL reservoir | soaked in cryo solution^[b]^ for 5 min |
| L6-9aa | 100 mM MES/Tris (6.5), 50 mM (NH_4_)_2_SO_4_, 10% PEG8000 | 1-2 µL protein^[a]^  1-2 µL reservoir | no treatment |
| L6-9aa  NADPH_4_ | 100 mM MES/Tris (6.5), 50 mM (NH_4_)_2_SO_4_, 12% PEG8000 | 1-2 µL protein^[a]^  1-2 µL reservoir | soaked in cryo solution^[b]^ for 18 min |
| L6-9aa  NADH_4_ | 100 mM MES/Tris (6.5), 50 mM (NH_4_)_2_SO_4_, 12% PEG8000 | 1-2 µL protein^[a]^  1-2 µL reservoir7 | soaked in cryo solution^[b]^ for 20 min |
| L6-[*Achr*OYE4]  NADPH_4_ | 100 mM Tris-HCl (7.5), 50 mM Na-tartrate, 8% PEG8000 | 1-2 µL protein^[a]^  1-2 µL reservoir  0.5 µL seeds (1:10) | soaked in reservoir solution^[c]^ for 120 min |
| L6-[*Achr*OYE4]  NADH_4_ | 100 mM Tris-HCl (7.5), 50 mM Na-tartrate, 8% PEG8000 | 1-2 µL protein^[a]^  1-2 µL reservoir  0.5 µL seeds (1:10) | soaked in reservoir solution^[c]^ for 120 min |

^[a]^ protein concentration was ~10 mg/mL. ^[b]^ Soaking in cryo solution (i.e., reservoir solution + 20% MPD) supplemented with cosubstrate powder. ^[c]^ Soaking in reservoir solution supplemented with cosubstrate powder.

**References**

Assil-Companioni L, Büchsenschütz HC, Solymosi D, Dyczmons-Nowaczyk NG, Bauer KKF, Wallner S, Macheroux P, Allahverdiyeva Y, Nowaczyk MM, Kourist R (2020) Engineering of NADPH supply boosts photosynthesis-driven biotransformations. ACS Catal. 10:11864–11877.

Böhmer S, Marx C, Goss R, Gilbert M, Sasso S, Happe T, Hemschemeier A (2023) *Chlamydomonas reinhardtii* mutants deficient for Old Yellow Enzyme 3 exhibit increased photooxidative stress. Plant Direct 7:e480.

Brigé A, Van Den Hemel D, Carpentier W, De Smet L, Van Beeumen JJ (2006) Comparative characterization and expression analysis of the four Old Yellow Enzyme homologues from *Shewanella oneidensis* indicate differences in physiological function. Biochem. J. 394:335–344.

Chaparro‐Riggers JF, Rogers TA, Vazquez‐Figueroa E, Polizzi KM, Bommarius AS (2007) Comparison of three enoate reductases and their potential use for biotransformations. Adv. Synth. Catal. 349:1521–1531.

Damada PH, Fraaije MW (2024) Identification of five robust novel ene-reductases from thermophilic fungi. Catalysts 14:764.

Damada PH, Rozeboom HJ, Fraaije MW (2025) Recombinant production and characterization of six ene‐reductases from *Penicillium steckii*. ChemBioChem 26:e202401007.

Fu Y, Castiglione K, Weuster‐Botz D (2013) Comparative characterization of novel ene‐reductases from cyanobacteria. Biotechnol. Bioeng. 110:1293–1301.

Fu Y, Hoelsch K, Weuster-Botz D (2012) A novel ene-reductase from Synechococcus sp. PCC 7942 for the asymmetric reduction of alkenes. Process Biochem. 47:1988–1997.

Iorgu AI, Hedison TM, Hay S, Scrutton NS (2019) Selectivity through discriminatory induced fit enables switching of NAD(P)H coenzyme specificity in Old Yellow Enzyme ene‐reductases. FEBS J. 286:3117–3128.

Kerschbaumer B, Totaro MG, Friess M, Breinbauer R, Bijelic A, Macheroux P (2024) Loop 6 and the β‐hairpin flap are structural hotspots that determine cofactor specificity in the FMN ‐dependent family of ene‐reductases. FEBS J. 291:1560–1574.

Knaus T, Paul CE, Levy CW, De Vries S, Mutti FG, Hollmann F, Scrutton NS (2016) Better than nature: nicotinamide biomimetics that outperform natural coenzymes. J. Am. Chem. Soc. 138:1033–1039.

Li Z, Wang Z, Meng G, Lu H, Huang Z, Chen F (2018) Identification of an ene reductase from Yeast *Kluyveromyces Marxianus* and application in the asymmetric synthesis of ( *R* )‐profen esters. Asian J. Org. Chem. 7:763–769.

Libardi SH, Ahmad A, Ferreira FB, Oliveira RJ, Caruso ÍP, Melo FA, De Albuquerque S, Cardoso DR, Burtoloso ACB, Borges JC (2024) Interaction between diterpene icetexanes and old yellow enzymes of Leishmania braziliensis and Trypanosoma cruzi. Int. J. Biol. Macromol. 259:129192.

Litthauer S, Gargiulo S, Van Heerden E, Hollmann F, Opperman DJ (2014) Heterologous expression and characterization of the ene-reductases from *Deinococcus radiodurans* and *Ralstonia metallidurans*. J. Mol. Catal. B Enzym. 99:89–95.

Mähler C, Kratzl F, Vogel M, Vinnenberg S, Weuster‐Botz D, Castiglione K (2019) Loop swapping as a potent approach to increase ene reductase activity with nicotinamide adenine dinucleotide (NADH). Adv. Synth. Catal. 361:2505–2513.

Ni Y, Yu H-L, Lin G-Q, Xu J-H (2014) An ene reductase from *Clavispora lusitaniae* for asymmetric reduction of activated alkenes. Enzyme Microb. Technol. 56:40–45.

Papadopoulou A, Peters C, Borchert S, Steiner K, Buller R (2022) Development of an ene reductase-based biocatalytic process for the production of flavor compounds. Org. Process Res. Dev. 26:2102–2110.

Pei X-Q, Xu M-Y, Wu Z-L (2016) Two "classical" Old Yellow Enzymes from Chryseobacterium sp. CA49: Broad substrate specificity of Chr-OYE1 and limited activity of Chr-OYE2. J. Mol. Catal. B Enzym. 123:91–99.

Richter N, Gröger H, Hummel W (2011) Asymmetric reduction of activated alkenes using an enoate reductase from *Gluconobacter oxydans*. Appl. Microbiol. Biotechnol. 89:79–89.

Robescu MS, Loprete G, Gasparotto M, Vascon F, Filippini F, Cendron L, Bergantino E (2022) The family keeps on growing: four novel fungal OYEs characterized. Int. J. Mol. Sci. 23:3050.

Robescu MS, Niero M, Hall M, Cendron L, Bergantino E (2020) Two new ene-reductases from photosynthetic extremophiles enlarge the panel of old yellow enzymes: CtOYE and GsOYE. Appl. Microbiol. Biotechnol. 104:2051–2066.

Scholtissek A, Ullrich SR, Mühling M, Schlömann M, Paul CE, Tischler D (2017) A thermophilic-like ene-reductase originating from an acidophilic iron oxidizer. Appl. Microbiol. Biotechnol. 101:609–619.

Sheng X, Yan M, Xu L, Wei M (2016) Identification and characterization of a novel Old Yellow Enzyme from *Bacillus subtilis* str.168. J. Mol. Catal. B Enzym. 130:18–24.

Singh Y, Sharma R, Mishra M, Verma PK, Saxena AK (2022) Crystal structure of ArOYE6 reveals a novel C‐terminal helical extension and mechanistic insights into the distinct class III OYEs from pathogenic fungi. FEBS J. 289:5531–5550.

Williams RE, Rathbone DA, Scrutton NS, Bruce NC (2004) Biotransformation of explosives by the Old Yellow Enzyme family of flavoproteins. Appl. Environ. Microbiol. 70:3566–3574.

Zhang Y, Teoh KH, Reed DW, Maes L, Goossens A, Olson DJH, Ross ARS, Covello PS (2008) The molecular cloning of artemisinic aldehyde Δ11(13) reductase and its role in glandular trichome-dependent biosynthesis of artemisinin in Artemisia annua. J. Biol. Chem. 283:21501–21508.

Zhang B, Zheng L, Lin J, Wei D (2016) Characterization of an ene-reductase from Meyerozyma guilliermondii for asymmetric bioreduction of α,β-unsaturated compounds. Biotechnol. Lett. 38:1527–1534.

Zhang B, Sun J, Zheng Y, Mao X, Lin J, Wei D (2022) Identification of a novel ene reductase from *Pichia angusta* with potential application in ( *R* )-levodione production. RSC Adv. 12:13924–13931.
